# Sexual behaviour among women using intramuscular depot medroxyprogesterone acetate, a copper intrauterine device, or a levonorgestrel implant for contraception: Data from the ECHO randomized trial

**DOI:** 10.1371/journal.pone.0299802

**Published:** 2024-05-09

**Authors:** G. Justus Hofmeyr, Mandisa Singata-Madliki, Joanne Batting, Petrus Steyn, Katherine K. Thomas, Rodal Issema, Ivana Beesham, Enough Mbatsane, Charles Morrison, Jen Deese, Jenni Smit, Neena Philip, Thesla Palanee-Phillips, Krishnaveni Reddy, Maricianah Onono, Timothy D. Mastro, Jared M. Baeten, on behalf of Evidence for Contraceptive Options and HIV Outcomes (ECHO) Trial Consortium

**Affiliations:** 1 University of Botswana, Gaborone, Botswana; 2 Effective Care Research Unit, University of the Witwatersrand/Walter Sisulu University, East London, South Africa; 3 Effective Care Research Unit, University of Witwatersrand, University of Fort Hare, East London, South Africa; 4 UNDP/UNFPA/UNICEF/WHO/World Bank Special Programme of Research, Development and Research Training in Human Reproduction, Geneva, Switzerland; 5 Department of Global Health, University of Washington, Seattle, Washington, United States of America; 6 Department of Epidemiology, University of Washington, Seattle, Washington, United States of America; 7 Faculty of Health Sciences, Department of Obstetrics and Gynaecology, MatCH Research Unit (MRU), University of the Witwatersrand, Durban, South Africa; 8 Setshaba Research Centre, Pretoria, South Africa; 9 FHI 360, Durham, North Carolina, United States of America; 10 Pfizer Inc., Collegeville, Pennsylvania, United States of America; 11 Mailman School of Public Health, ICAP at Columbia University, New York, New York, United States of America; 12 Wits Reproductive Health and HIV Institute, School of Public Health, University of the Witwatersrand, Johannesburg, Gauteng, South Africa; 13 Kenya Medical Research Institute, Centre for Microbiology Research, Kisumu, Kenya; 14 Gilead Sciences, Foster City, California, United States of America; Johns Hopkins University Bloomberg School of Public Health, UNITED STATES

## Abstract

**Background:**

Contraceptive use has complex effects on sexual behaviour and mood, including those related to reduced concerns about unintended pregnancy, direct hormonal effects and effects on endogenous sex hormones. We set out to obtain robust evidence on the relative effects of three contraceptive methods on sex behaviours, which is important for guiding contraceptive choice and future contraceptive developments.

**Methods:**

This is a secondary analysis of data from the Evidence for Contraceptive Options and HIV Outcomes (ECHO) randomized trial in which 7,829 HIV-uninfected women from 12 sites in Eswatini, Kenya, South Africa and Zambia seeking contraception were randomly assigned to intramuscular depot-medroxyprogesterone acetate (DMPA-IM), the copper intrauterine device (Cu-IUD) or the levonorgestrel (LNG) implant. Data collected for 12 to 18 months using 3-monthly behavioural questionnaires that relied on recall from the preceding 3 months, were used to estimate relative risk of post-baseline sex behaviours, as well as sexual desire and menstrual bleeding between randomized groups using modified Poisson regression.

**Results:**

We observed small but generally consistent effects wherein DMPA-IM users reported lower prevalence of specified high risk sexual behaviours than implant users than Cu-IUD users (the ‘>‘ and ‘<‘ symbols indicate statistically significant differences): multiple sex partners 3.6% < 4.8% < 6.2% respectively; new sex partner 3.0% < 4.0% <5.3%; coital acts 16.45, 16.65, 17.12 (DMPA-IM < Cu-IUD); unprotected sex 65% < 68%, 70%; unprotected sex past 7 days 33% <36%, 37%; sex during vaginal bleeding 7.1%, 7.1% < 8.9%; no sex acts 4.1%, 3.8%, 3.4% (DMPA-IM > Cu-IUD); partner has sex with others 10% < 11%, 11%. The one exception was having any sex partner 96.5%, 96.9% < 97.4% (DMPA-IM < Cu-IUD). Decrease in sexual desire was reported by 1.6% > 1.1% >0.5%; amenorrhoea by 49% > 41% >12% and regular menstrual pattern by 26% <35% < 87% respectively.

**Conclusions:**

These findings suggest that women assigned to DMPA-IM may have a modest decrease in libido and sexual activity relative to the implant, and the implant relative to the Cu-IUD. We found more menstrual disturbance with DMPA-IM than with the implant (and as expected, both more than the Cu-IUD). These findings are important for informing the contraceptive choices of women and policymakers and highlight the need for robust comparison of the effects of other contraceptive methods as well.

## Introduction

Contraceptive use has complex effects on sexual behaviour [1] and mood, including those related to reduced concerns about unintended pregnancy, direct hormonal effects and effects on endogenous sex hormones. Hormonal contraceptive use has been associated with reduced sexual function [2]. Method switching and discontinuation has been attributed to reduced desire experienced during hormonal contraception [3]. In a previous randomized trial, reduced sexual activity was found among participants randomized to injectable progestogens versus the copper-bearing IUD (Cu-IUD) [4,5]. Injectable progestogen use has been associated with lower libido, hypothesized to be a result of hypoestrogenic effects of medroxyprogesterone [6].

Hormonal effects on menstruation have also been associated with switching and discontinuation of contraceptive method [7]. There is evidence of an increased susceptibility to sexually transmitted infection during menstruation and South African [8] and North American [9] studies have suggested that coitus during menstruation may be a risk factor for HIV transmission. Some have postulated divergent effects of hormonal contraception on HIV acquisition risk, including both effects increasing susceptibility (e.g., immune suppression, hypoestrogenism) and those reducing exposure (e.g., reduced sexual activity due to reduced libido and during menstruation) [10,11].

Robust evidence on the relative side effects of alternative methods is important for guiding contraceptive choice and for development of future contraceptive options.

The Evidence for Contraceptive Options and HIV Outcomes (ECHO) Trial [12] was designed to address a decades-long concern, based on data from observational studies that hormonal contraceptives, particularly DMPA-IM, may increase HIV acquisition risk [13–15]; the trial did not find a statistically significant difference in the risk of HIV acquisition across three contraceptive methods.

The current analysis tests whether longitudinal sex behaviours, sexual desire or menstrual pattern differs in women using the three methods of contraception offered in the ECHO Trial.

## Materials and methods

This is a secondary analysis of data from the ECHO Trial. The trial methods have been reported previously [12,16]. Briefly, 7829 HIV negative young women from 12 sites in, Eswatini, Kenya, South Africa and Zambia aged 16–35 years seeking contraception were allocated in a 1:1:1 ratio in centrally administered random sequence to DMPA-IM, the Cu-IUD or the LNG implant, and followed after one month then three-monthly for 12 to 18 months. The three-monthly visits included behavioural questionnaires covering the 3-month interval prior to the visit; those data were self-reported. As previously reported, retention and method continuation in the trial were high, with women completing 94% of scheduled study visits and using their randomized method for 92% of person-time.

Our primary interest was to compare post-randomization sex behaviours and related outcomes by randomization group, overall and over time. The analysis plan for this work was completed before starting the analyses. Analyses were performed using R version 4.1 (R Core Team 2021).

### Outcomes

Sex behaviour outcomes identified a priori were self-reported (for the past 3 months, unless otherwise specified): having any sex partner, having multiple sex partners, having a new sex partner, number of coital acts (continuous variable), sex during a time of vaginal bleeding, any unprotected sex, and, for the past 7 days, number of unprotected sex acts (both as a continuous variable and dichotomized as any unprotected sex). We also analyzed no sex acts, partner having sex with other partners, and measures of side effects: reduced sexual desire, regular menstruation and absence of menstruation.

#### Pairwise comparisons between randomized groups over all follow-up

To compare each behaviour by randomized group in an overall comparison, we made pairwise comparisons between randomized groups of each outcome in an intention-to-treat (ITT) analysis, meaning women’s visits were analysed according to randomized assignment rather than by whether they were adhering to their randomized method. We used all available post-baseline observations. For binary outcomes, we estimated risk ratios (RR) since many outcomes were common. We used log Poisson models modified by using robust errors; robust errors allow valid inference in the context of a non-Poisson error structure arising from a binary rather than count outcome [17] as well as adjusting model standard errors to account for correlation in outcomes arising from analyzing multiple observations per woman. For count outcomes (number of coital acts, number of unprotected sex acts), we estimated incidence rate ratios (IRRs), using a Poisson model with robust standard errors to account for correlation within outcomes due to analyzing multiple observations per woman. Models were adjusted for study site and the baseline value of the behaviour, to account for any existing differences by group at baseline.

#### Pairwise comparisons between randomized groups in trends over time

To examine changes in behaviours over time we focused on early differences, as reported for the first quarter after study start (0–3 month behaviour), and later differences, as reported for the quarter ending 12 months after study start (9–12 month behaviour). Comparisons used the same models as were used to analyse the overall study time period, above, except that robust standard errors were not used for count outcomes since one time point was modelled in each regression.

#### Age

Pairwise comparisons of behaviours were examined for effect modification by baseline subgroup of age (<25 vs > = 25 years) by testing interaction terms between subgroup and randomised group in the regression model with p-value generated using likelihood ratio tests.

#### Continuous use

To examine whether any observed differences in the ITT analysis varied when limited to periods of continuous use, we performed the analyses as above but limited to time when the woman was continuously using the allocated contraceptive method. We defined “continuously using” as was done in the ECHO primary paper [12].

#### Multiple testing and power

The level of statistical significance regarded as meaningful was not adjusted for multiple comparisons, as each outcome was expected to measure a different aspect of sexual behaviour and repeated measurements over time were addressed within the overall analysis which included all timepoints and adjusted confidence intervals accordingly. However, in a study of 7829 women powered to detect differences in HIV acquisition, we likely had very high power to detect even small differences which may not necessarily be clinically significant.

### Ethical review

Ethical approval was obtained from the Protection of Human Subjects Committee of FHI 360 (approval number: 523201–146) and from the Ethics Committee of the World Health Organization (approval numbers: A65897 and A65922). Each participating site also obtained approval from appropriate local Institutional Review Boards [12]. All participants provided written informed consent, which included information about randomisation, each study contraceptive method, and their rights as research participants. Women aged 16–17 years were enrolled only in settings where permissible by national regulations and local ERC approval for their self-consent.

## Results

Women attended study visits between 14 December 2015 and 31 October 2018. Baseline demographic and behavioural data were similar between the three groups, as expected in a large randomized trial (Table 1). Most women were aged 18–30 years (90%), never married (80%), had at least secondary schooling (90%) and had had a prior pregnancy (81%). Prior contraceptive use was high among study participants including 51% who had used DMPA-IM, 22% norethisterone enanthate 200mg intramuscular (NET-EN), 12% who had used oral contraceptives, and 6% who had used contraceptive implants. Few women (0.8%) had previously used an IUD.

**Table 1:** Baseline characteristics, by randomized group, N (%) or median (Q1, Q3).

Characteristics^a^	DMPA-IM (n = 2609)	LNG Implant (n = 2613)	Cu-IUD (n = 2607)	All (n = 7829)
**Age (years)**				
16–17	17 (0.7%)	21 (0.8%)	26 (1.0%)	64 (0.8%)
18–20	695 (27%)	683 (26%)	684 (26%)	2,062 (26%)
21–24	953 (37%)	956 (37%)	913 (35%)	2,822 (36%)
25–30	719 (28%)	735 (28%)	752 (29%)	2,206 (28%)
31+	225 (8.6%)	218 (8.3%)	232 (8.9%)	675 (8.6%)
**Marital status**				
Never Married	2,087 (80%)	2,085 (80%)	2,090 (80%)	6,262 (80%)
Married	502 (19%)	503 (19%)	504 (19%)	1,509 (19%)
Previously Married	20 (0.8%)	25 (1.0%)	13 (0.5%)	58 (0.7%)
**Lives with partner**	763 (29%)	760 (29%)	773 (30%)	2,296 (30%)
**Education**				
No schooling	16 (0.6%)	21 (0.8%)	12 (0.5%)	49 (0.6%)
Primary school	216 (8.3%)	260 (10.0%)	247 (9.5%)	723 (9.2%)
Secondary school	1,967 (75%)	1,918 (73%)	1,930 (74%)	5,815 (74%)
Post-secondary school	410 (16%)	414 (16%)	418 (16%)	1,242 (16%)
**BMI**	25.0 (21.8, 30.0)	25.5 (22.1, 30.5)	25.2 (21.8, 30.1)	25.3 (21.9, 30.2)
**Any prior pregnancy**	2,100 (80%)	2,146 (82%)	2,121 (81%)	6,367 (81%)
**Condom used last time had sex**	97 (7.3%)	109 (7.9%)	111 (8.0%)	317 (7.7%)
*Sex behaviors in last 3 months*				
**Any sex partners**	2,603 (99.8%)	2,605 (99.7%)	2,600 (99.7%)	7,808 (99.7%)
**More than one sex partner**	173 (6.6%)	165 (6.3%)	198 (7.6%)	536 (6.8%)
**Any new partner**	122 (4.7%)	106 (4.1%)	131 (5.0%)	359 (4.6%)
**Total number of coital acts**	9 (4, 20)	9 (4, 20)	9 (4, 20)	9 (4, 20)
**Any unprotected sex**	1890 (73%)	1904 (73%)	1907 (73%)	5,701 (73%)
**Any sex for money**	32 (1.2%)	27 (1.0%)	30 (1.2%)	89 (1.1%)
**Sex during vaginal bleeding**	247 (9.5%)	273 (10%)	272 (10%)	792 (10%)
**Partner has sex with others**				
Yes	383 (15%)	418 (16%)	435 (17%)	1,236 (16%)
No	886 (34%)	831 (32%)	854 (33%)	2,571 (33%)
Don’t know	1,325 (51%)	1,349 (52%)	1,309 (50%)	3,983 (51%)

^a^ Eligibility requirements excluded participants who used DMPA, Implant, Cu-IUD, or NET-EN in the 6 months prior to enrolment.

While at enrolment nearly all participants had had a sex partner during the previous 3 months and most women (73%) had had unprotected sex, few women had had multiple partners (7%), new sex partners (5%), sex for money (1%) or sex during vaginal bleeding (10%). The median number of coital acts during the previous 3 months was 9.

During the study period, women continued to be sexually active, rarely reporting no partners (<4% of quarters in all groups) and averaging 16–17 sex acts per quarter, but also rarely reporting new partners (≤5% of quarters) or multiple partners (< = 6% of quarters) (Table 2). Report of at least one incidence of unprotected sex was fairly common (65–70% at some point within the quarter, and 33–37% within in the past 7 days, with mean in the past 7 days of one unprotected act). Comparisons of outcomes between each pair of randomized groups, by ITT are also shown in Table 2. Many comparisons were statistically significant, even when differences were small, as expected when comparing large groups.

**Table 2:** Statistical comparisons of sex behaviours, sexual desire, partner behaviour and menstruation over the previous 3 months (or where stated, 7 days) by randomized group throughout follow-up, N (%) or mean (standard deviation), intention to treat analysis.

	DMPA-IM, (n = 13216)^a^	Cu-IUD, (n = 13769)^a^	LNG Implant, (n = 13871)^a^	DMPA-IM vs Cu-IUD		DMPA-IM vs LNG Implant		Cu-IUD vs LNG Implant	
				Adjusted RR or IRR (95% CI)^b^	p-value^b^	Adjusted RR or IRR (95% CI)^b^	p-value^b^	Adjusted RR or IRR (95% CI)^b^	p-value^b^
**Sex behaviours**									
Any sex partner	12758 (96.5%)	13408 (97.4%)	13442 (96.9%)	0.99 (0.98, 1.00)	<0.001	1.00 (0.99, 1.00)	0.155	1.01 (1.00, 1.01)	0.033
More than one sex partners	478 (3.6%)	865 (6.3%)	679 (4.9%)	0.58 (0.49, 0.68)	<0.001	0.75 (0.63, 0.89)	0.001	1.30 (1.13, 1.51)	<0.001
New sex partner	394 (3.0%)	733 (5.3%)	550 (4.0%)	0.57 (0.49, 0.67)	<0.001	0.77 (0.65, 0.91)	0.002	1.35 (1.17, 1.56)	<0.001
Total coital acts	16.45 (17.18)	17.12 (18.04)	16.65 (17.02)	0.96 (0.92, 1.00)	0.029	0.98 (0.94, 1.02)	0.280	1.02 (0.99, 1.06)	0.232
Unprotected sex acts (past 7 days)	1.03 (1.76)	0.96 (1.70)	0.96 (1.49)	1.09 (1.01, 1.17)	0.021	1.08 (1.01, 1.15)	0.028	0.99 (0.93, 1.06)	0.800
Any unprotected sex acts (past 7 days)	4403 (33%)	5084 (37%)	5023 (36%)	0.91 (0.87, 0.95)	<0.001	0.92 (0.88, 0.96)	<0.001	1.01 (0.97, 1.05)	0.647
Any unprotected sex acts	8630 (65%)	9605 (70%)	9492 (68%)	0.94 (0.92, 0.97)	<0.001	0.96 (0.93, 0.98)	<0.001	1.01 (0.99, 1.04)	0.283
Sex during vaginal bleeding	939 (7.1%)	1238 (8.9%)	981 (7.1%)	0.80 (0.72, 0.89)	<0.001	1.01 (0.91, 1.13)	0.802	1.27 (1.14, 1.41)	<0.001
No sex acts (vs any sex, with or without condoms)	539 (4.1%)	471 (3.4%)	522 (3.8%)	1.21 (1.04, 1.41)	0.011	1.09 (0.94, 1.26)	0.274	0.89 (0.77, 1.04)	0.140
**Side effects and partner behaviour**									
Decrease in sexual desire	207 (1.6%)	76 (0.5%)	155 (1.1%)	2.81 (2.13, 3.70)	<0.001	1.39 (1.09, 1.76)	0.007	0.49 (0.37, 0.66)	<0.001
Partner has sex with others (vs no or don’t know)	1267 (10%)	1561 (11%)	1546 (11%)	0.86 (0.78, 0.96)	0.006	0.86 (0.78, 0.96)	0.005	1.00 (0.90, 1.10)	0.978
Menstruation pattern no bleeding (vs all other options)	6496 (49%)	1282 (9.2%)	5685 (41%)	5.11 (4.69, 5.56)	<0.001	1.18 (1.14, 1.23)	<0.001	0.23 (0.21, 0.25)	<0.001
Menstruation pattern regular (vs all other patterns)	3397 (26%)	10783 (78%)	4888 (35%)	0.33 (0.32, 0.35)	<0.001	0.74 (0.70, 0.78)	<0.001	2.22 (2.15, 2.30)	<0.001

^a^ Statistics presented: n (%); Mean (SD).

^b^ Adjusted RRs and p-values computed for binary outcomes and adjusted IRRs and p-values for count outcomes (i.e., sex acts) with modified Poisson regression with robust standard errors, adjusted for enrollment site and the same behaviour at baseline.

### DMPA-IM versus the Cu-IUD

Reporting at least one unprotected sex act was observed to be less common in the DMPA-IM group than the Cu-IUD group (65% vs 70%, risk ratio (RR) 0.94, 95% confidence interval (CI) 0.92 to 0.97, p<0.001). Any unprotected sex during the past 7 days showed a similar pattern (33% vs 37%; RR 0.91, 0.87 to 0.95; p<0.001), although the mean number of unprotected sex acts in the past 7 days was slightly higher in the DMPA-IM group than in the Cu-IUD group (1.03 vs 0.96, IRR 1.09, 1.01 to 1.17, p = 0.021). All other sexual exposure variables indicated statistically significant but modestly lower risk behaviour in the DMPA-IM than the Cu-IUD group: any sex partner (96.5% vs 97.4%; RR 0.99, 95% CI 0.98 to 1.00; p<0.001); multiple sex partners (3.6% vs 6.3%; RR 0.58, 0.49 to 0.68; p<0.001); new sex partner (3.0% vs 5.3%; RR 0.57, 0.49 to 0.67; p<0.001); mean count of total coital acts (16.5 vs 17.1; IRR 0.96, 0.92 to 1.00; p = 0.03); and sex during vaginal bleeding (7.1% vs 8.9%; RR 0.80, 0.72 to 0.89; p <0.001).

Decrease in sexual desire was rare in all groups but was more common with DMPA-IM than the Cu-IUD (1.6% vs. 0.5%; RR = 2.81, 2.13 to 3.70; p<0.001). As expected, absence of menstruation was far more common in the DMPA than the Cu-IUD group (49% vs. 9.2%; RR 5.11, 4.69 to 5.56; p<0.001). Women reporting that their partner has sex with others was less common with DMPA-IM than with the Cu-IUD use (9.6% vs. 11.3%, RR 0.86, 0.78 to 0.96; p = 0.006).

### DMPA-IM versus the LNG implant

Group differences in sexual behaviours between DMPA-IM and LNG Implant were generally smaller than between DMPA-IM and Cu-IUD, and were statistically significantly different for fewer behaviours. Those which were different generally showed lower levels of behaviours associated with STI risk in the DMPA-IM group: in the past 3 months, report of unprotected sex was modestly lower with DMPA-IM compared to LNG implant (65% vs 68%, RR 0.96, 0.93 to 0.98; p<0.001) as was the proportion with unprotected sex reported for the past 7 days (33% vs. 36%; RR = 0.92, 0.88 to 0.96; p<0.001); while the number of unprotected sex acts in the same timeframe was slightly higher (1.03 vs. 0.96. IRR 1.08, 1.01 to 1.15. p = 0.028). Multiple sex partners (3.6% vs 4.9%; RR 0.75, 0.63 to 0.89; p = 0.001); and new sex partner (3.0% vs 4.0%; RR 0.77, 0.65 to 0.91; p = 0.002) were also lower with DMPA-IM. There were no significant differences for report of having a sex partner (96.5% vs 96.9%; RR 1.00, 0.99 to 1.00; p = 0.16); mean number of coital acts (16.5 vs 16.7; IRR 0.98, 0.94 to 1.02; p = 0.28); or sex during menstruation (7.1% vs 7.1%; RR 1.01, 0.91 to 1.13; p = 0.80).

Decrease in sexual desire was more common with DMPA-IM than the LNG implant (1.6% vs 1.1%; RR 1.39, 1.09 to 1.76, p = 0.007), as was absence of menstruation (49% vs 41%, RR1.18, 1.14 to 1.23, p<0.001), both with smaller estimated differences than between DMPA-IM and Cu-IUD. Report of partner having sex with others was lower for DMPA-IM (9.6% vs. 11.2%; RR 0.86, 0.78 to 0.96; p = 0.005).

### Cu-IUD versus the LNG implant

Behaviours associated with STI risk were generally modestly higher for the Cu-IUD group than for the LNG group, with statistically significant differences for, in the past 3 months, having a sex partner (97.4% vs 96.9%; RR 1.01, 1.00 to 1.01; p = 0.03); multiple sex partners (6.3% vs 4.9%; RR 1.30, 1.13 to 1.31; p <0.001); new sex partner (5.3% vs 4.0%; RR 1.35, 1.17 to 1.56; p <0.001; and sex during vaginal bleeding (8.99% vs 7.1%; RR 1.27, 1.14 to 1.41; p <0.001). However, unprotected sex in the past 3 months was similar in the two groups (70% vs 68%, RR 1.01, 0.99 to 1.04; p = 0.28), as was any unprotected sex in the past 7 days (37% vs 36%; RR 1.01, 0.97 to 1.01; p = 0.65); number of unprotected sex acts in past 7 days (0.96 vs 0.96, 0.99 (0.93, 1.06), p = 0.80); and mean coital acts (17.1 vs 16.7; IRR 1.02, 0.99 to 1.06; p = 0.23).

Decrease in sexual desire was less common in the Cu-IUD than the LNG group (0.5% vs 1.1%; RR 0.49, 0.37 to 0.66; p<0.001) as was absence of menstruation (9% vs. 41%; RR = 0.23, 0.21 to 0.25; p<0.001). Report of partner having sex with others was similar (11% vs. 11%; RR 1.0, 0.90 to 1.10; p = 0.98).

### Comparisons within age subgroups, during continuous use, and over time

About a third of women were at least 25 years old, and results of comparisons by randomized group in this subgroup of women were similar to those in women < 25 years old (S1 Table). One difference was found to be statistically significant: having multiple sex partners for women with DMPA-IM vs with LNG implant had statistically different RRs by age subgroup, with women <25 showing a stronger effect of having fewer partners on DMPA-IM relative to LNG implant than women 25+ years (3% vs 5%, RR 0.64 (0.51, 0.79) in women <25, and 4% vs 5%, RR0.96 (0.72, 1.28) in women 25+ years). However, a total of 24 tests for effect modification by age were performed, making at least one spurious association likely. This single finding did not appear to be part of a pattern of difference in older vs younger women.

Sexual behaviour data for women limited to time periods when women were continuously using their contraceptive method (i.e., did not switch methods) were very similar to the ITT data (S2 Table).

**Fig 1:**
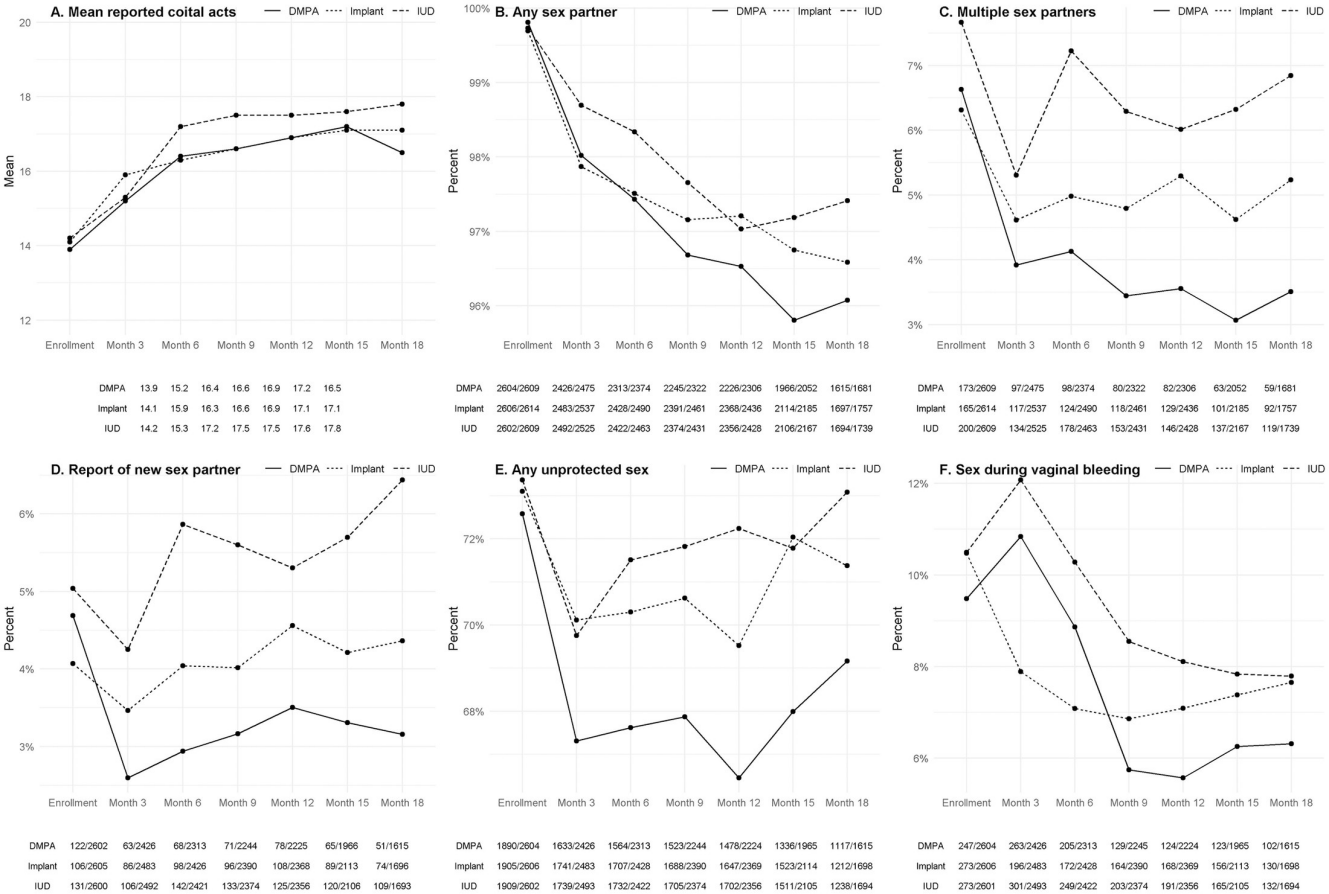
Plots of sex behaviours showing relationships over time, enrollment to month 18.

**Fig 2:**
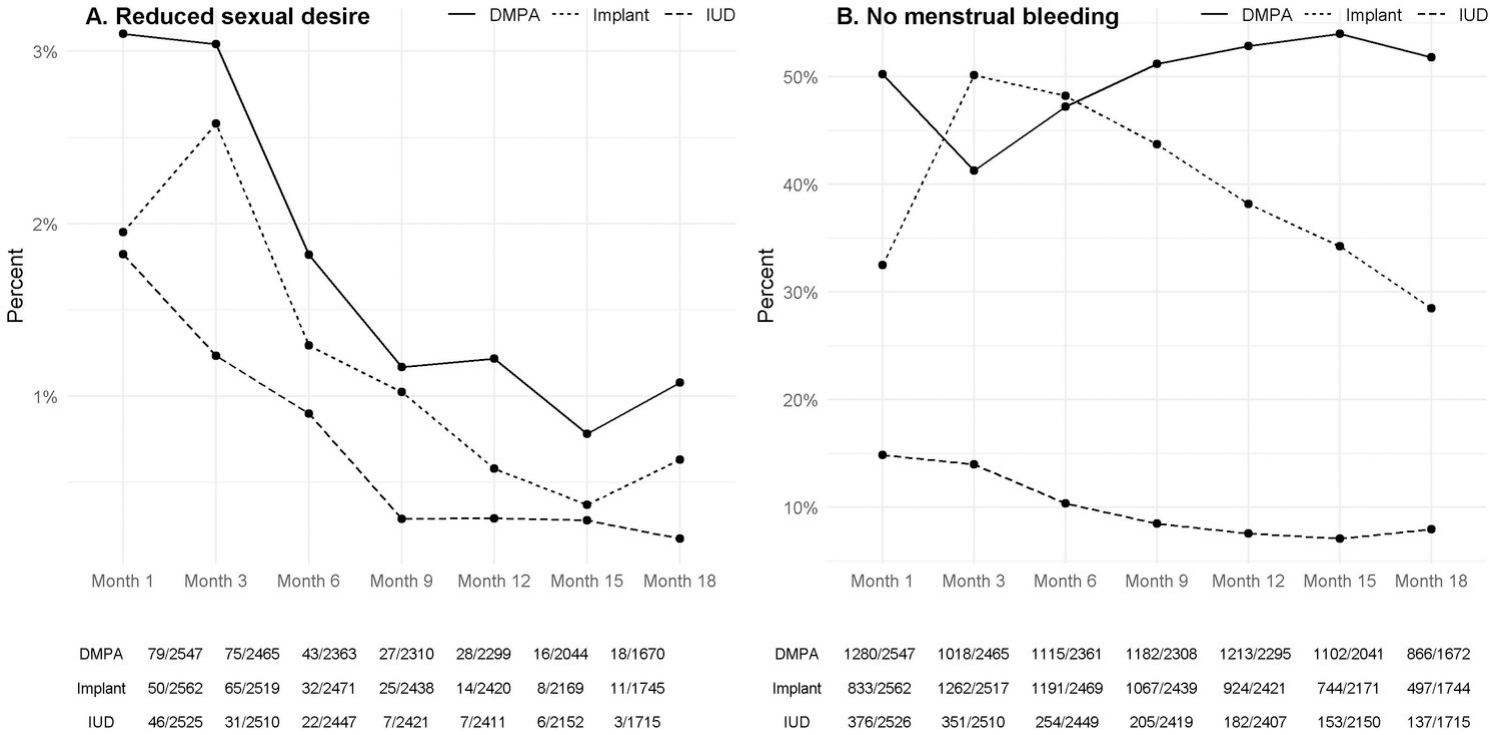
Plots of side effect variables showing relationships over time, month 1 to month 18.

Plots of sex behaviours showing relationships over time are shown in Fig 1. Any unprotected sex declined sharply from baseline, possibly reflecting counselling in an HIV prevention trial, but declined most sharply in the DMPA-IM group and remained lower throughout follow-up, with similar levels whether comparing groups at the first follow-up visit, or later in the trial (S3 Table). Plots of side effects (reduced sexual desire and no menstrual bleeding) over time are shown in Fig 2.

## Discussion

The ECHO Trial provided a unique opportunity to compare effects of three contraceptive methods within the context of a stringent randomized trial.

### Behaviour and libido

These data suggest that women assigned to DMPA-IM had modestly reduced sexual activity when compared to women assigned to Cu-IUD and the LNG implant and both DMPA-IM and the LNG implant compared to the Cu-IUD. The magnitudes of effects were generally small, and statistical significance was bolstered by the very large sample size of the trial. Nevertheless, the data provide important information for women’s contraceptive choices and for consideration for the development of novel contraceptive agents. The findings are consistent with those of a previous pragmatic randomized trial which found less sexual activity among women randomized to injectable progestogen contraception than to the Cu-IUD [4,5].

The possibility that this may be related to reduced libido is supported by the finding in this study of significantly more reporting of decreased sexual desire in the DMPA-IM group compared to the Cu-IUD or the LNG group. This is biologically plausible because of the known effects of DMPA-IM on suppression of endogenous estrogen and testosterone, both of which are considered to enhance libido. Ancillary hormonal studies of the ECHO Trial at the East London, South Africa site found lower estradiol levels at 6 months in women randomized to DMPA-IM than those randomized to the Cu-IUD and the LNG implant, and the implant group were significantly lower than with the Cu-IUD [18]. Free testosterone was significantly lower with DMPA-IM than with the LNG implant, with the Cu-IUD being intermediate. (GJ Hofmeyr, personal communication). Thus, the lower sexual desire with DMPA-IM compared with the other two groups is consistent with the lower estradiol and free testosterone with DMPA-IM.

There was modestly lower sexual exposure with the implant than with the Cu-IUD.

### Menstruation

We observed as was expected, that regular menstruation and thus the potential for sex during menstruation was more common with the Cu-IUD than with DMPA-IM or the LNG implant. We have shown for the first time in the context of a randomized comparison that absence of menstruation is more common and regular menstruation less common with DMPA-IM than with the LNG implant.

### Relevance to HIV acquisition

Whether the small but generally consistent differences in sexual behaviour may have contributed to the overall similar HIV risk among the three methods found in the ECHO Trial cannot be confirmed from this analysis. Secondary comparisons of HIV incidence among the three contraceptive methods using causal models showed essentially no effect from adjusting for follow-up behaviours of condomless sex, having a new partner or having multiple partners on comparisons between randomized contraceptive method, implying that the differences in those behaviours by method do not contribute to differences in HIV risk by method [12].

### Limitations

Data were reliant on participants’ recall from the previous three months, or for some outcomes, seven days. Recalled behavioural data are subject to recall bias. However, in a well-conducted randomized study, recall bias should affect all groups equally, thus the relative differences between methods should be reliable. In addition, the recall data are consistent with objective data on semen exposure from an ancillary study at three of the ECHO sites. Prostate-specific antigen levels in cervical samples were less frequent in a sample of women allocated to DMPA-IM than to the Cu-IUD, and significantly less than the implant [19].

Multiple end-point testing carries an inherent risk of sporadic spurious differences occurring by chance. However, chance significant differences are less likely with a large sample size as in this study, and the considerable consistency in results across all the endpoints suggests that the findings are generally robust.

The study included two progestogen (DMPA-IM and LNG implant) and one non-hormonal contraceptive method; the findings cannot be extrapolated to other methods. This study has highlighted the fact that progestogen contraceptives may differ considerably and unpredictably in their effects on behaviour and menstruation and cannot be considered similar in terms of behavioural effects and effects on HIV risk. For example, the etonorgestrel implant has been associated with impaired sexual function, thought to be related to suppression of estrogen and testosterone [20], but we do not have robust data comparing this effect with other methods. Intramuscular norethisterone enanthate has many similar behavioural effects to DMPA-IM, but less glucocorticoid and immunosuppressive effects] [21,22]. This might confer a similar reduction in exposure without increased immune susceptibility and thus lower net risk of HIV. Robust randomized trials are needed to compare the many and complex effects of various contraceptive methods to provide a sound basis on which women and policy-makers can make choices. While our study does not include qualitative data, ancillary studies conducted a single ECHO Trial site have examined sexual behaviour, mood and menstruation in more detail [23,24].

### Strengths

This study is unique with respect to the large sample size, robust randomized methodology, in-depth counselling about HIV risk and the provided contraceptive side-effects and high rates of adherence and follow-up which are hallmarks of the ECHO Trial.

## Conclusions

Sexual desire, sexual exposure behaviours, occurrence of menstruation and regular menstruation were generally lower in women randomized to DMPA-IM than to the Cu-IUD, to DMPA-IM than to the LNG implant, and to the LNG implant than to the Cu-IUD. Sex during menstruation followed the same pattern, except that it was similar between DMPA-IM and the LNG implant. These findings are important for informing the contraceptive choices of women and policy-makers and the counselling offered by clinicians, and highlight the need for robust comparison of the effects of other contraceptive methods.

## Supporting information

S1 Table Statistical comparisons of sex behaviors by randomized group in subgroups by age, throughout follow-up, ITT. (DOCX)

S2 Table Statistical comparisons of sex behaviors by randomized group throughout follow-up, during continuous use. (DOCX)

S3 Table Statistical comparison of sex behaviors by randomized group, early and later effects, intention to treat analysis. (DOCX)
