# Supplementary material for: Sexual behaviour among women using intramuscular depot medroxyprogesterone acetate, a copper intrauterine device, or a levonorgestrel implant for contraception: Data from the ECHO randomized trial
Source: PLoS One. 2024 May 9;19(5):e0299802. doi: 10.1371/journal.pone.0299802 (PMC11081305; doi:10.1371/journal.pone.0299802)
Supplement: S1 Table — (DOCX) [file pone.0299802.s001.docx]

**Supplemental Table S1.** Statistical comparisons of sex behaviours by randomized group in subgroups by age, throughout follow-up, ITT.

|  |  |  |  |  |  |  | **Relative Risk or IRR***^2^* |  |  |  |
| --- | --- | --- | --- | --- | --- | --- | --- | --- | --- | --- |
| **Behaviour** | **Age** | **DMPA-IM (n = 13216)^a^** | **Cu-IUD (n = 13769)^a^** | **LNG Implant (n = 13871)^a^** | **DMPA-IM vs Cu-IUD^b^** | **p-value^b^** | **DMPA-IM vs LNG Implant^b^** | **p-value^b^** | **Cu-IUD vs LNG Implant^b^** | **p-value^b^** |
| Any sex partner |  |  |  |  |  | 0.791 |  | 0.856 |  | 0.791 |
|  | <25 | 8044 (96%) | 8268 (97%) | 8467 (96%) | 0.99 (0.98, 1.00) |  | 1.00 (0.99, 1.00) |  | 1.01 (1.00, 1.01) |  |
|  | 25+ | 4714 (97%) | 5140 (98%) | 4975 (98%) | 0.99 (0.98, 1.00) |  | 0.99 (0.99, 1.00) |  | 1.00 (1.00, 1.01) |  |
| Multiple sex partners |  |  |  |  |  | 0.828 |  | 0.023 |  | 0.828 |
|  | <25 | 264 (3%) | 476 (6%) | 448 (5%) | 0.57 (0.46, 0.70) |  | 0.64 (0.51, 0.79) |  | 1.12 (0.94, 1.35) |  |
|  | 25+ | 214 (4%) | 389 (7%) | 231 (5%) | 0.58 (0.45, 0.75) |  | 0.96 (0.72, 1.28) |  | 1.65 (1.29, 2.10) |  |
| New sex partners |  |  |  |  |  | 0.922 |  | 0.142 |  | 0.922 |
|  | <25 | 235 (3%) | 431 (5%) | 365 (4%) | 0.56 (0.46, 0.69) |  | 0.70 (0.57, 0.86) |  | 1.24 (1.04, 1.48) |  |
|  | 25+ | 159 (3%) | 302 (6%) | 185 (4%) | 0.57 (0.44, 0.73) |  | 0.90 (0.68, 1.19) |  | 1.58 (1.25, 2.01) |  |
| Total coital acts |  |  |  |  |  | 0.404 |  | 0.856 |  | 0.404 |
|  | <25 | 15.21 (15.92) | 15.53 (16.30) | 15.19 (16.42) | 0.97 (0.93, 1.02) |  | 0.98 (0.94, 1.03) |  | 1.01 (0.96, 1.06) |  |
|  | 25+ | 18.56 (18.95) | 19.67 (20.27) | 19.12 (17.71) | 0.94 (0.88, 1.00) |  | 0.97 (0.92, 1.03) |  | 1.04 (0.98, 1.10) |  |
| Total unprotected sex acts (past 7 days) |  |  |  |  |  | 0.195 |  | 0.264 |  | 0.195 |
|  | <25 | 1.02 (1.34) | 1.01 (1.48) | 1.00 (1.46) | 1.04 (0.96, 1.11) |  | 1.04 (0.96, 1.11) |  | 1.00 (0.93, 1.08) |  |
|  | 25+ | 1.04 (2.25) | 0.90 (1.96) | 0.90 (1.53) | 1.16 (1.00, 1.34) |  | 1.13 (0.99, 1.28) |  | 0.97 (0.85, 1.11) |  |
| Any unprotected sex acts (past 7 days) |  |  |  |  |  | 0.185 |  | 0.126 |  | 0.185 |
|  | <25 | 2577 (31%) | 2813 (33%) | 2834 (32%) | 0.94 (0.88, 1.00) |  | 0.95 (0.89, 1.01) |  | 1.01 (0.95, 1.07) |  |
|  | 25+ | 1826 (38%) | 2271 (43%) | 2189 (43%) | 0.88 (0.82, 0.94) |  | 0.88 (0.82, 0.95) |  | 1.00 (0.94, 1.07) |  |
| Any unprotected sex acts |  |  |  |  |  | 0.882 |  | 0.624 |  | 0.882 |
|  | <25 | 5296 (63%) | 5754 (67%) | 5765 (66%) | 0.94 (0.91, 0.97) |  | 0.96 (0.93, 0.99) |  | 1.02 (0.99, 1.05) |  |
|  | 25+ | 3334 (69%) | 3851 (73%) | 3727 (73%) | 0.95 (0.91, 0.99) |  | 0.95 (0.91, 0.99) |  | 1.00 (0.96, 1.04) |  |
| Any sex during vaginal bleeding |  |  |  |  |  | 0.383 |  | 0.614 |  | 0.383 |
|  | <25 | 602 (7%) | 753 (9%) | 628 (7%) | 0.83 (0.73, 0.95) |  | 1.04 (0.91, 1.19) |  | 1.25 (1.10, 1.43) |  |
|  | 25+ | 337 (7%) | 485 (9%) | 353 (7%) | 0.75 (0.63, 0.90) |  | 0.98 (0.80, 1.19) |  | 1.30 (1.09, 1.55) |  |
| *^a^ Statistics presented: n (%); Mean (SD)* | | | | | | | | | | |
| *^b^ RRs and p-values computed for binary outcomes and IRRs and p-values for count outcomes (i.e., sex acts) with modified Poisson regression with robust standard errors, adjusted for enrollment site. P-values indicate whether RRs, or IRRs, comparing randomized groups differ by age* | | | | | | | | | | |
