# Supplementary material for: Sexual behaviour among women using intramuscular depot medroxyprogesterone acetate, a copper intrauterine device, or a levonorgestrel implant for contraception: Data from the ECHO randomized trial
Source: PLoS One. 2024 May 9;19(5):e0299802. doi: 10.1371/journal.pone.0299802 (PMC11081305; doi:10.1371/journal.pone.0299802)
Supplement: S2 Table — (DOCX) [file pone.0299802.s002.docx]

**Supplemental Table S2.** Statistical comparisons of sex behaviours by randomized group throughout follow-up, during continuous use.

|  |  |  |  |  |  | **Relative Risk or IRR***^2^* |  |  |  |
| --- | --- | --- | --- | --- | --- | --- | --- | --- | --- |
| **Behavior** | **DMPA-IM (n = 9378)^a^** | **Cu-IUD (n = 8902)^a^** | **LNG Implant (n = 9742)^a^** | **DMPA-IM vs Cu-IUD^b^** | **p-value^b^** | **DMPA-IM vs LNG Implant^b^** | **p-value^b^** | **Cu-IUD vs LNG Implant^b^** | **p-value^b^** |
| Any sex partner | 9123 (97%) | 8703 (98%) | 9445 (97%) | 0.99 (0.99, 1.00) | 0.071 | 1.00 (1.00, 1.01) | 0.367 | 1.01 (1.00, 1.02) | 0.006 |
| Multiple sex partners | 330 (4%) | 542 (6%) | 469 (5%) | 0.59 (0.48, 0.72) | <0.001 | 0.73 (0.59, 0.90) | 0.004 | 1.24 (1.03, 1.49) | 0.021 |
| New sex partners | 255 (3%) | 466 (5%) | 382 (4%) | 0.54 (0.44, 0.66) | <0.001 | 0.70 (0.57, 0.87) | <0.001 | 1.30 (1.09, 1.55) | 0.004 |
| Total coital acts | 17.66 (17.91) | 16.37 (16.39) | 16.18 (17.02) | 1.00 (0.95, 1.04) | 0.926 | 1.02 (0.97, 1.06) | 0.434 | 1.02 (0.98, 1.06) | 0.366 |
| Total unprotected sex acts (past 7 days) | 1.02 (1.84) | 0.94 (1.43) | 0.94 (1.46) | 1.13 (1.04, 1.23) | 0.003 | 1.13 (1.04, 1.22) | 0.005 | 0.99 (0.92, 1.07) | 0.892 |
| Any unprotected sex acts (past 7 days) | 3339 (36%) | 3261 (37%) | 3511 (36%) | 0.92 (0.87, 0.97) | 0.003 | 0.93 (0.89, 0.99) | 0.014 | 1.02 (0.96, 1.07) | 0.550 |
| Any unprotected sex acts | 6295 (67%) | 6192 (70%) | 6617 (68%) | 0.94 (0.91, 0.97) | <0.001 | 0.96 (0.93, 0.99) | 0.019 | 1.02 (0.99, 1.05) | 0.144 |
| Any sex during vaginal bleeding | 652 (7%) | 772 (9%) | 623 (6%) | 0.78 (0.69, 0.89) | <0.001 | 1.05 (0.91, 1.20) | 0.518 | 1.34 (1.17, 1.53) | <0.001 |
| Decrease in sexual desire | 144 (2%) | 47 (1%) | 98 (1%) | 2.72 (1.93, 3.83) | <0.001 | 1.41 (1.05, 1.89) | 0.022 | 0.52 (0.36, 0.75) | <0.001 |
| Partner has sex with others (vs no or don’t know) | 874 (9%) | 1069 (12%) | 1098 (11%) | 0.79 (0.70, 0.90) | <0.001 | 0.81 (0.72, 0.93) | 0.002 | 1.03 (0.91, 1.16) | 0.664 |
| Menstruation pattern no bleeding (vs all other options) | 4859 (52%) | 639 (7%) | 4197 (43%) | 7.02 (6.22, 7.94) | <0.001 | 1.19 (1.14, 1.25) | <0.001 | 0.17 (0.15, 0.19) | <0.001 |
| Menstruation pattern regular (vs all other patterns) | 2244 (24%) | 7347 (83%) | 3364 (35%) | 0.29 (0.28, 0.31) | <0.001 | 0.70 (0.65, 0.74) | <0.001 | 2.40 (2.30, 2.50) | <0.001 |
| *^a^ Statistics presented: n (%); Mean (SD)* | | | | | | | | | |
| *^b^ RRs and p-values computed for binary outcomes and IRRs and p-values for count outcomes (i.e., sex acts) with modified Poisson regression with robust standard errors, adjusted for enrollment site.* | | | | | | | | | |
