# Supplementary material for: Sexual behaviour among women using intramuscular depot medroxyprogesterone acetate, a copper intrauterine device, or a levonorgestrel implant for contraception: Data from the ECHO randomized trial
Source: PLoS One. 2024 May 9;19(5):e0299802. doi: 10.1371/journal.pone.0299802 (PMC11081305; doi:10.1371/journal.pone.0299802)
Supplement: S3 Table — (DOCX) [file pone.0299802.s003.docx]

**Supplemental Table S3.** Statistical comparison of sex behaviours by randomized group, early and later effects, intention to treat analysis

| **Follow-up Risk Behaviour Previous 3 Months^a^** | **DMPA-IM vs Cu-IUD** | **p-value** | **DMPA-IM vs LNG Implant** | **p-value** | **Cu-IUD vs LNG Implant** | **p-value** |
| --- | --- | --- | --- | --- | --- | --- |
| ***Early effect (3 months)*** |  |  |  |  |  |  |
| Any sex partner | 0.99 (0.99, 1.00) | 0.058 | 1.00 (0.99, 1.01) | 0.695 | 1.01 (1.00, 1.02) | 0.022 |
| Multiple sex partners | 0.74 (0.58, 0.96) | 0.022 | 0.86 (0.66, 1.11) | 0.245 | 1.15 (0.91, 1.46) | 0.252 |
| New sex partners | 0.62 (0.45, 0.84) | 0.002 | 0.76 (0.55, 1.04) | 0.085 | 1.22 (0.93, 1.61) | 0.155 |
| Total coital acts | 1.00 (0.94, 1.06) | 0.926 | 0.96 (0.91, 1.02) | 0.211 | 0.97 (0.91, 1.02) | 0.246 |
| Total unprotected sex acts (past 7 days) | 1.12 (1.00, 1.26) | 0.060 | 1.03 (0.92, 1.15) | 0.592 | 0.92 (0.82, 1.03) | 0.150 |
| Any unprotected sex acts (past 7 days) | 0.90 (0.83, 0.97) | 0.004 | 0.92 (0.86, 1.00) | 0.037 | 1.03 (0.96, 1.10) | 0.429 |
| Any unprotected sex acts | 0.96 (0.93, 1.00) | 0.048 | 0.96 (0.93, 1.00) | 0.035 | 1.00 (0.96, 1.03) | 0.898 |
| Any sex during vaginal bleeding | 0.88 (0.76, 1.03) | 0.119 | 1.35 (1.13, 1.61) | <0.001 | 1.53 (1.29, 1.81) | <0.001 |
| ***Late effect (12 months)*** |  |  |  |  |  |  |
| Any sex partner | 1.00 (0.99, 1.01) | 0.407 | 0.99 (0.98, 1.00) | 0.238 | 1.00 (0.99, 1.01) | 0.721 |
| Multiple sex partners | 0.60 (0.46, 0.78) | <0.001 | 0.68 (0.52, 0.89) | 0.005 | 1.14 (0.91, 1.43) | 0.260 |
| New sex partners | 0.66 (0.50, 0.87) | 0.004 | 0.77 (0.58, 1.03) | 0.077 | 1.17 (0.91, 1.50) | 0.228 |
| Total coital acts | 0.96 (0.91, 1.01) | 0.101 | 0.99 (0.94, 1.04) | 0.714 | 1.03 (0.98, 1.09) | 0.198 |
| Total unprotected sex acts (past 7 days) | 1.08 (0.96, 1.22) | 0.185 | 1.07 (0.97, 1.19) | 0.185 | 0.99 (0.88, 1.12) | 0.890 |
| Any unprotected sex acts (past 7 days) | 0.94 (0.88, 1.01) | 0.088 | 0.96 (0.89, 1.03) | 0.258 | 1.02 (0.95, 1.09) | 0.561 |
| Any unprotected sex acts | 0.92 (0.89, 0.96) | <0.001 | 0.96 (0.92, 0.99) | 0.019 | 1.04 (1.00, 1.07) | 0.041 |
| Any sex during vaginal bleeding | 0.69 (0.55, 0.85) | <0.001 | 0.80 (0.64, 0.99) | 0.044 | 1.16 (0.95, 1.41) | 0.148 |

*^a^ RRs and p-values computed for binary outcomes and IRRs and p-values for count outcomes (i.e., sex acts) with modified Poisson regression with robust standard errors, adjusted for enrollment site.*
