# Supplementary material for: Sexual behaviour among women using intramuscular depot medroxyprogesterone acetate, a copper intrauterine device, or a levonorgestrel implant for contraception: Data from the ECHO randomized trial
Source: PLoS One. 2024 May 9;19(5):e0299802. doi: 10.1371/journal.pone.0299802 (PMC11081305; doi:10.1371/journal.pone.0299802)
Supplement: Attachment — Submitted filename: Response to Reviewers_13Dec22.pdf [file pone.0299802.s004.pdf]

Thank you for reviewing our manuscript and providing valuable feedback which we believe will strengthen the manuscript. We have responded to each of the reviewer's comments in blue coloured font below. Changes in the manuscript have been tracked and page and line numbers have been provided.

Journal Requirements:

When submitting your revision, we need you to address these additional requirements.

1. Please ensure that your manuscript meets PLOS ONE's style requirements, including those for file naming. The PLOS ONE style templates can be found at https://journals.plos.org/plosone/s/file?id=wjVg/PLOSOne_formatting_sample_main_body.pdf and

https://journals.plos.org/plosone/s/file?id=ba62/PLOSOne_formatting_sample_title_authors_affiliations.pdf

Thank you for raising this. We have formatted the paper as per the journal requirements, including those for file naming.

2. You indicated that you had ethical approval for your study. In your Methods section, please ensure you have also stated whether you obtained consent from parents or guardians of the minors included in the study or whether the research ethics committee or IRB specifically waived the need for their consent.

Thank you for this comment. We have added the following line to the ethical review section under the methods which states these women self-consented:

"Women aged 16-17 were enrolled only in settings where permissible by national regulations and local ERC approval for their self-consent." (Page 10, L206-208)

Reviewer #1: This paper reports secondary analysis of a large three-arm randomized trial. Women were randomized to DMPA-IM, the Cu-IUD, or LNG implant.

Minor revisions:

1- Abstract, Methods: The abstract states that the relative risk was estimated. List the events or provide a general term for events in which relative risks were estimated.

Thank you for noting this omission. The sentence in the abstract now reads (added text highlighted):

Data collected for 12 to 18 months using 3-monthly behavioural questionnaires that relied on recall from the preceding 3 months, were used to estimate relative risk of sex behaviours between randomized groups using modified Poisson regression. (Page 4, L82-85)

2- Cite the statistical software used for the analysis.

Thank you for requesting this; we now cite the software, which was R version 4.1. (Page 7, L148-149)

3- Indicate the date range subjects participated in the study.

We have added the date range during which participants participated in the study, which was from 14 December 2015 to 31 October 2018. (Page 10, L211)

4- Table 2: Clearly indicate that particular columns of p-values are for pairwise comparisons.

We have reorganized the table header to more clearly show that both RR/IRRs and p values arise from pairwise comparisons. (Pages 13-15)

5- Include Supplemental Figure S1 as a standard figure within the manuscript.

Thank you for this suggestion, we now include the figure as a standard figure in our manuscript. (Page 19)
