# Supplementary material for: Sexual behaviour among women using intramuscular depot medroxyprogesterone acetate, a copper intrauterine device, or a levonorgestrel implant for contraception: Data from the ECHO randomized trial
Source: PLoS One. 2024 May 9;19(5):e0299802. doi: 10.1371/journal.pone.0299802 (PMC11081305; doi:10.1371/journal.pone.0299802)
Supplement: Attachment — Submitted filename: Responses to reviewers_Sex Beh manuscript_18Dec23.pdf [file pone.0299802.s005.pdf]

Due to the discussions around the ECHO trial, we have tried to get more feedback for the manuscript. We have received feedback from another reviewer since your last response to reviewers. We would be grateful if you can address the new reviewer comments as well.

This is noted. Please find responses to editor's and reviewers' comments below. We have provided page and line numbers in the revised manuscript where indicated.

**Comments from editor**

I have a couple of points to make.

1. There have been pairwise comparisons of each of the arms with a risk ratio and p value provided. I wonder about the need for such hypothesis testing since there are no base levels (true controls). Presenting just the n, incidence measure with 95% CI would be just as effective in providing the estimates from a well done RCT. As you have rightly pointed out multiple times, the large sample sizes and small event rates provide exaggerated risk ratios which may not be clinically significant.

Thank you for this comment. We very much agree with the editor that the descriptive data should be emphasized, so that statistical significance is not taken to imply clinical significance for participants. This is why, given limited space in the abstract, we give descriptive statistics of each outcome by randomized group and not the risk ratios.

We submit that the large sample size and small event rates do not yield exaggerated risk ratios (risk ratios would not be expected to be biased by either large sample sizes or small event rates); our intention was to point out that the large sample size means we can find statistically significant differences which are small enough that they are not clinically significant for participants. We reviewed our statements carefully to ensure we do not imply bias, in Lines 207-209 (Page 10):

> "However, in a study of 7829 women powered to detect differences in HIV acquisition, we likely had very high power to detect even small differences which may not necessarily be clinically significant."

And Lines 247-248 (Page 13):

> "Many comparisons were statistically significant, even when differences were small, as expected when comparing large groups."

Regarding whether RRs should be computed at all, we don't agree that only a 'true control' comparator would justify hypothesis testing. We think that comparison across different highly effective contraceptive methods is an important study question and does justify hypothesis testing. Our hypothesis, which perhaps we have not been clear enough in stating (see our response to reviewer 2, below) is that there are differences among the studied highly effective contraceptive choices regarding how they affect sexual behaviours and related outcomes. We believe this is important, particularly for women who want highly effective contraceptive options and may benefit from information to inform choices. We believe we have balanced using statistical testing with careful interpretation and highlighting the numbers compared rather than just RRs.

2. In the discussion, a mention has been made of the inability to comment on HIV acquisition using the present study. Has there been an effort to work with mediation analyses to provide more insight into the contribution of the changes in sexual behaviours towards HIV acquisition?

The primary publication of the ECHO trial presented secondary analyses comparing HIV acquisition by randomized group using causal methods. The causal models were conducted in two ways; first, using models did not adjust for any follow-up covariates, and second, adjusting for post-baseline sex behaviours of condomless sex, having a new partner, and having multiple partners (Table S10 in the

primary publication's supplement, and provided below) Comparison of results of these models (rows 2 and 3 below) shows that the adjustment for post-baseline differences in sex behaviors, which is what we report on in the current manuscript, made little to no difference to relative risks of HIV acquisition between groups. This implies that observed differences in sex behaviors caused by differing contraceptive methods do not translate to differences in HIV risk. We now explain this more clearly in the discussion under Relevance to HIV acquisition: (Page 21, Lines 370-377):

> "Whether the small but generally consistent differences in sexual behaviour may have contributed to the overall similar HIV risk among the three methods found in the ECHO Trial cannot be confirmed from this analysis. **Secondary comparisons of HIV incidence among the three contraceptive methods using causal models showed essentially no effect from adjusting for follow-up behaviours of condomless sex, having a new partner or having multiple partners on comparisons between randomized contraceptive method, implying that the differences in those behaviours by method do not contribute to differences in HIV risk by method [12]."** *(added text in bold)*

For reference, Table S10 from the ECHO primary publication:

| | DMPA-IM vs Copper IUD | | DMPA-IM vs LNG mplant | | Copper IUD vs LNG Implant | |
|---|---|---|---|---|---|---|
| **Analysis** | **HR (96% CI)** | **p-value** | **HR (96% CI)** | **p-value** | **HR (96% CI)** | **p-value** |
| Unweighted, adjusted for baseline covariates | 1.17 (0.89, 1.53) | 0.25 | 1.34 (1.02, 1.77) | 0.029 | 1.15 (0.88, 1.51) | 0.29 |
| Weighted, adjusted for baseline covariates | 1.07 (0.82, 1.40) | 0.62 | 1.27 (0.96, 1.68) | 0.080 | 1.19 (0.91, 1.56) | 0.19 |
| Weighted, adjusted for baseline and time-varying covariates | 1.10 (0.84, 1.44) | 0.49 | 1.29 (0.97, 1.71) | 0.060 | 1.18 (0.90, 1.55) | 0.22 |

Continuous use estimates included only time on study with continuous use of randomized methods, as defined in the Methods, and included data from 7317 women. Causal analysis methods were used to estimate HRs during continuous use, with inverse probability weighting for discontinuation of randomized method, adjusted for baseline covariates (age ≤24, having living children, living with husband or primary partner, vaginal sex without a condom, more than one sex partner, and new sex partner) and time-varying covariates (vaginal sex without a condom, more than one sex partner and new sex partner in the previous 3 months). Inverse probability weights for discontinuation of randomized method relied on vaginal sex without a condom, vaginal sex last 7 days, and having primary partner in the previous 3 months. Covariate data for calculating weights were missing for 82 (1.1%) of women; and covariates for model adjustment were missing for another 9 (0.1%) of women, corresponding to 0.1% of visits. Women missing censoring weights or covariate data were excluded from the causal analysis.

## Comments from reviewers

Reviewer #2: (No Response)
Noted.

Reviewer #4: Thank you for the opportunity to review this very interesting paper. The paper is generally well written and addresses an important issue in public health- the effects of contraceptives on sexual desire (mood), adverse events and sexual behavior. The authors used data from the ECHO trial which has courted controversy since its design, conduct and dissemination of results. While a lot of the controversy regarding DMPA-IM and HIV acquisition has decreased, this contraceptive method still carries stigma of forced use, lack of education and options. The authors need to make it clear what their hypothesis going into the study was and better discuss what the implications of these findings are.
Thank you for these comments. We have defined our hypothesis more clearly, and the implications of our study findings in the comments below.

I have made more specific comments by section.

• Abstract, background: the authors need to add a hypothesis for this analysis. I am struggling to understand why this analysis is necessary given that the data used is from a randomized trial. If the randomization at baseline worked well, should we expect differences in sexual behavior, libido, bleeding and sexual behavior by arm?

The reason we might expect to find differences is that DMPA and Levonorgestrel have large and different effects on endogenous hormone levels including estradiol, testosterone and sex hormone binding globulin, which are associated with sexual function and mood. Our hypothesis was that there may be differences in sexual activity across different highly effective contraceptive methods studied. We believe this is of interest for women making choices among contraceptive options. We have edited the abstract to explain our study aim more clearly (Page 4, Lines 73-76):

> "**We set out to obtain** robust evidence on the relative effects of **three contraceptive** methods **on sex behaviours, which** is important for guiding contraceptive choice and future contraceptive developments." (*added text in bold*)

Regarding why we set out to compare sexual differences in a randomized trial, the purpose of randomization is to obtain comparable groups at baseline, for both measured and unmeasured factors. There is no reason to expect that measurements after baseline (i.e., once on the different treatments) will not differ between groups depending on the effect of the treatment(s) on the outcome(s) of interest, and this is what we set out to examine. As stated in the methods (Page 8, Lines 168-172):

> "To compare each behaviour by randomized group in an overall comparison, we made pairwise comparisons between randomized groups of each outcome in an intention-to-treat (ITT) analysis, meaning women's visits were analysed according to randomized assignment rather than by whether they were adhering to their randomized method. We used all available **post-baseline** observations." (*bold added here for emphasis*)

We have now specified this more clearly in the abstract (Page 4, Lines 83-86):

> "Data collected for 12 to 18 months using 3-monthly behavioural questionnaires that relied on recall from the preceding 3 months, were used to estimate relative risk of **post-baseline** sex behaviours, as well as sexual desire and menstrual bleeding between randomized groups using modified Poisson regression." (*relevant new text in bold*)

And, at the start of the methods (Page 7, Lines 149-151)

> "Our primary interest was to compare **post-randomization sex** behaviours and related outcomes by randomization group, overall and over time. The analysis plan for this work was completed before starting the analyses." (*relevant new text in bold*)

• Abstract, methods: The authors need to specify the sexual behaviors in question

While we'd like to list these in the abstract methods as well as in the results, we examined a variety of sexual behaviours, and it would make the abstract considerably longer to list the sexual behaviours we examined in both methods and when providing the results. However, to improve clarity in the methods we now clarify that we also examined sexual desire and menstrual bleeding, as the reviewer's point elsewhere that these are not sex behaviours, is well taken. (Page 4, Lines 83-86)

> "Data collected for 12 to 18 months using 3-monthly behavioural questionnaires that relied on recall from the preceding 3 months, were used to estimate relative risk of post-baseline sex behaviours, **as well as sexual desire and menstrual bleeding** between randomized groups using modified Poisson regression." (*relevant new text in bold*)

• Abstract, results: The authors report that DMPA-IM was associated with lower sexual behaviors. I am not sure if lower is the correct term. Lower prevalence of specified high risk sexual behaviours maybe would be better.

This point is noted. We have edited as suggested (Page 4, Lines 88-90):

> "We observed small but generally consistent effects wherein DMPA-IM users **reported lower prevalence of specified high risk sexual behaviours** than implant users than Cu-IUD users …" (*relevant new text in bold*)

• Abstract results: Are libido and menstrual disturbances sexual behaviours? I would have thought these would be adverse effects of the contraception and would be expected to explain some of the sexual behaviours then observed. This is the reason I am asking the authors to state a hypothesis and add libido and menstrual disturbances as objectives/ outcomes in the analysis.

We appreciate this point and now specify "sex behaviours, sexual desire and menstrual bleeding" in the abstract methods. (Page 4, Lines 83-86)

> "Data collected for 12 to 18 months using 3-monthly behavioural questionnaires that relied on recall from the preceding 3 months, were used to estimate relative risk of **post-baseline** sex behaviours, **as well as sexual desire and menstrual bleeding** between randomized groups using modified Poisson regression." (*new text in bold*)

We also have moved the results of these two outcomes to a separate sentence rather than including them in the sentence giving results for sex behaviours: (Pages 4-5, Lines 88-99)

> "We observed small but generally consistent effects wherein DMPA-IM users had reported lower prevalence of specified high risk sexual behaviours than implant users than Cu-IUD users (the '>' and '<' symbols indicate statistically significant differences): multiple sex partners 3.6% < 4.8% < 6.2% respectively; new sex partner 3.0% < 4.0% <5.3%; coital acts 16.45, 16.65, 17.12 (DMPA-IM < Cu-IUD); unprotected sex 65% < 68%, 70%; unprotected sex past 7 days 33% <36%, 37%; sex during vaginal bleeding 7.1%, 7.1% < 8.9%; no sex acts 4.1%, 3.8%, 3.4% (DMPA-IM > Cu-IUD); partner has sex with others 10% < 11%, 11%. The one exception was having any sex partner 96.5%, 96.9% <97.4% (DMPA-IM < Cu-IUD). **Decrease in sexual desire was reported by 1.6% > 1.1% >0.5%; amenorrhoea by 49% > 41% >12% and regular menstrual pattern by 26% <35% <87% respectively."** (*relevant new text in bold)*

• Can the authors also present the relative risks in addition to the raw percentages

While we would have liked to present the relative risks in the abstract in addition to the raw percentages, with so many outcomes of interest, and with 3 pairwise comparisons for each one, with limited abstract space, we have had to limit the detail reported for each comparison. We are also cognizant of the editor's point#1 asking us to emphasize descriptives over relative risks. Therefore, while we appreciate the reviewer's interest in seeing these highlighted, we have kept the text as is for the abstract.

<u>Introduction</u>

• Line 133- can the authors specify the behaviors in question and state the hypothesis

We now state the hypothesis of the current analysis in the final sentence of the introduction, as follows: (Page 7, Lines 134-136)

> "The current analysis **tests whether** longitudinal **sex** behaviour**s, sexual desire or** menstrual **pattern differs in** women **using** the three methods of contraception offered in the ECHO Trial." (*new text in bold*)

Thank you for your query about specifying the behaviours tested. We listed the outcomes in the methods under "Outcomes", but the list is now more precisely detailed for clarity: (Page 8, Lines 154-164)

> "**Sex behaviour** outcomes **identified a priori** were self-reported (for the past 3 months, unless otherwise specified): having any sex partner, having multiple sex partners, having a new sex partner, number of coital acts (continuous variable), sex during a time of vaginal bleeding, any unprotected sex, and, for the past 7 days, number of unprotected sex acts (both as a continuous variable and dichotomized as any unprotected sex). **We also analyzed no sex acts, partner having sex with other partners, and measures of side effects: reduced sexual desire, regular menstruation and absence of menstruation."** (relevant new text in bold)

Checking Table 2 (Pages 14-16), we found that Table 2 erroneously reported the same outcome twice under two different names, "Any unprotected sex acts" and "sex but not always used condom". We have removed the latter from Table 2 to avoid redundancy. We have also revised the labelling in Table 2 to avoid calling all outcomes 'Behaviours" and now separate those that are side effects or a behaviour of the woman's partner, not the woman.

Methods:

• Line 151- 157: see earlier comment regarding side effects vs behaviors

See response to immediately prior to this comment.

• Line 171: Did the authors also consider adjusting for age, living with partner and any other variables for which there appeared to be imbalance at baseline e.g. new partner at baseline, and more than one partner in the last 3 months at baseline

Age was very balanced at baseline, as was the proportion living with a partner, so we did not adjust for these variables. For each outcome, we adjusted for the baseline value of that outcome in the same woman to ensure that we account for any baseline imbalance. We now clarify this further under "Pairwise comparisons between randomized groups over all follow-up": (Page 9, Lines 182-183)

> "Models were adjusted for study site and the baseline value of the behaviour, **to account for any existing differences by group at baseline.**" (*new relevant text in bold*)

<u>Results</u>

Table 1:

• please present baseline data on menstrual disturbances if that data is available

Unfortunately, menstrual disturbance was monitored as a side effect and only collected after baseline/enrolment, when participants would be on their methods, so we are unable to add it to the baseline data in Table 1. We did collect it at M1 (month 1) and we include that data in the longitudinal figure (Figure 2) requested further below.

• Are the differences in any new partner and more than one sex partner by arm significant?

We did not statistically test for differences in baseline characteristics due to the fact that we already know that any differences between groups are due to chance; calculating the probability of the observed difference being due to chance alone, (i.e., p-value) wouldn't answer a clear question here. However, the reviewer has pointed out baseline differences here that are important to adjust for in our analyses of post-baseline data. Even with large randomized groups, when comparing many

characteristics, we have a few which do not end up closely balanced, and as we have high power to detect small differences between groups, small differences already in place at baseline should be adjusted for. We have adjusted all our comparisons for the baseline value of the same variable, as described in response to this reviewer's query about line 171 above and now clearly stated in the footnote to Table 2 (see below).

Table 2:

• in the main analysis, a 50% difference in HIV incidence was the threshold for recommending policy change, is there such a threshold for the differences in adverse events such as menstrual disturbances, libido and sexual behavior

Thank you for raising this point. For this analysis, we did not pre-set such a threshold as we were not computing a sample size.

• Indicate that RR are adjusted

Thank you for pointing out this gap, we have clarified in the table columns and the footnote that the models are adjusted (Pages 14-16)

> "[b] RRs and p-values computed for binary outcomes and IRRs and p-values for count outcomes (i.e., sex acts) with modified Poisson regression with robust standard errors, **adjusted for enrollment site and the same behaviour at baseline."** (new relevant text in bold)

Figure 1

• Plot also menstrual disturbances and libido

We have added 'Figure 2' to present these, which were both monitored as side effects. The reviewer will note that unlike the sex behaviours, we do not have data at enrolment, before the woman began her contraceptive, but we did collect data at month 1 (M1) and we included that in the figure.

<u>Discussion</u>

• Was there any qualitative work done in this trial or elsewhere on the psychological effects of absent or irregular menstruation could explain effects of DMPA-IM on libido and sexual behaviors.

We have referenced ancillary studies which have examined sexual behaviour, mood and menstruation in more detail in a single-centre sub-set of the ECHO Trial. (Page 22, Lines 404-406)

> "While our study does not include qualitative data, ancillary studies conducted a single ECHO Trial site have examined sexual behaviour, mood and menstruation in more detail (23, 24)"

• I feel that the so what of this paper needs to be strengthened. What are we saying to women who opt for DMPA-IM and the health care providers that provide it?

We have expanded the conclusion as follows (Page 23, Lines 417-420):

> "These findings are important for informing the contraceptive choices of women and policy-makers **and the counselling offered by clinicians**, and highlight the need for robust comparison of the effects of other contraceptive methods." (*added text in bold*)
