# Supplementary material for: Sexual behaviour among women using intramuscular depot medroxyprogesterone acetate, a copper intrauterine device, or a levonorgestrel implant for contraception: Data from the ECHO randomized trial
Source: PLoS One. 2024 May 9;19(5):e0299802. doi: 10.1371/journal.pone.0299802 (PMC11081305; doi:10.1371/journal.pone.0299802)
Supplement: Peer review history

# Peer review: Decision Letter 0

**DOI:** 10.1371/journal.pone.0299802.r001

**Authors:** Maria Elisabeth Johanna Zalm

31 Oct 2022

PONE-D-22-20307Sexual behaviour among women using intramuscular depot medroxyprogesterone acetate, a copper intrauterine device, or a levonorgestrel implant for contraception: data from the ECHO randomized trialPLOS ONE

Dear Dr. Beesham,

Thank you for submitting your manuscript to PLOS ONE. After careful consideration, we feel that it has merit but does not fully meet PLOS ONE’s publication criteria as it currently stands. Therefore, we invite you to submit a revised version of the manuscript that addresses the points raised during the review process. Your manuscript has been assessed by a statistical reviewer and their comments are appended below.  The reviewer comments that your study would benefit from additional detail and clarification regarding your data analysis in the manuscript.  Could you please carefully revise the manuscript to address all comments raised?

Please note that we have only been able to secure a single reviewer to assess your manuscript. We are issuing a decision on your manuscript at this point to prevent further delays in the evaluation of your manuscript. Please be aware that the editor who handles your revised manuscript might find it necessary to invite additional reviewers to assess this work once the revised manuscript is submitted. However, we will aim to proceed on the basis of this single review if possible. 

Please submit your revised manuscript by Dec 13 2022 11:59PM. If you will need more time than this to complete your revisions, please reply to this message or contact the journal office at plosone@plos.org. When you're ready to submit your revision, log on to https://www.editorialmanager.com/pone/ and select the 'Submissions Needing Revision' folder to locate your manuscript file.

Please include the following items when submitting your revised manuscript:

- A rebuttal letter that responds to each point raised by the academic editor and reviewer(s). You should upload this letter as a separate file labeled 'Response to Reviewers'.
- A marked-up copy of your manuscript that highlights changes made to the original version. You should upload this as a separate file labeled 'Revised Manuscript with Track Changes'.
- An unmarked version of your revised paper without tracked changes. You should upload this as a separate file labeled 'Manuscript'.

If you would like to make changes to your financial disclosure, please include your updated statement in your cover letter. Guidelines for resubmitting your figure files are available below the reviewer comments at the end of this letter.

If applicable, we recommend that you deposit your laboratory protocols in protocols.io to enhance the reproducibility of your results. Protocols.io assigns your protocol its own identifier (DOI) so that it can be cited independently in the future. For instructions see: https://journals.plos.org/plosone/s/submission-guidelines#loc-laboratory-protocols. Additionally, PLOS ONE offers an option for publishing peer-reviewed Lab Protocol articles, which describe protocols hosted on protocols.io. Read more information on sharing protocols at https://plos.org/protocols?utm_medium=editorial-email&utm_source=authorletters&utm_campaign=protocols.

We look forward to receiving your revised manuscript.

Kind regards,

Maria Elisabeth Johanna Zalm, Ph.D

Editorial Office

PLOS ONE

Journal Requirements:

When submitting your revision, we need you to address these additional requirements.

1. Please ensure that your manuscript meets PLOS ONE's style requirements, including those for file naming. The PLOS ONE style templates can be found at https://journals.plos.org/plosone/s/file?id=wjVg/PLOSOne_formatting_sample_main_body.pdf and 

https://journals.plos.org/plosone/s/file?id=ba62/PLOSOne_formatting_sample_title_authors_affiliations.pdf

2. You indicated that you had ethical approval for your study. In your Methods section, please ensure you have also stated whether you obtained consent from parents or guardians of the minors included in the study or whether the research ethics committee or IRB specifically waived the need for their consent.

[Note: HTML markup is below. Please do not edit.]

Reviewers' comments:

Reviewer's Responses to Questions

**Comments to the Author**

1. Is the manuscript technically sound, and do the data support the conclusions?

The manuscript must describe a technically sound piece of scientific research with data that supports the conclusions. Experiments must have been conducted rigorously, with appropriate controls, replication, and sample sizes. The conclusions must be drawn appropriately based on the data presented. 

Reviewer #1: Yes

**********

2. Has the statistical analysis been performed appropriately and rigorously? 

Reviewer #1: Yes

**********

3. Have the authors made all data underlying the findings in their manuscript fully available?

The PLOS Data policy requires authors to make all data underlying the findings described in their manuscript fully available without restriction, with rare exception (please refer to the Data Availability Statement in the manuscript PDF file). The data should be provided as part of the manuscript or its supporting information, or deposited to a public repository. For example, in addition to summary statistics, the data points behind means, medians and variance measures should be available. If there are restrictions on publicly sharing data—e.g. participant privacy or use of data from a third party—those must be specified.

Reviewer #1: Yes

**********

4. Is the manuscript presented in an intelligible fashion and written in standard English?

PLOS ONE does not copyedit accepted manuscripts, so the language in submitted articles must be clear, correct, and unambiguous. Any typographical or grammatical errors should be corrected at revision, so please note any specific errors here.

Reviewer #1: Yes

**********

5. Review Comments to the Author

Please use the space provided to explain your answers to the questions above. You may also include additional comments for the author, including concerns about dual publication, research ethics, or publication ethics. (Please upload your review as an attachment if it exceeds 20,000 characters)

Reviewer #1: This paper reports secondary analysis of a large three-arm randomized trial. Women were randomized to DMPA-IM, the Cu-IUD, or LNG implant.

Minor revisions:

1- Abstract, Methods: The abstract states that the relative risk was estimated. List the events or provide a general term for events in which relative risks were estimated.

2- Cite the statistical software used for the analysis.

3- Indicate the date range subjects participated in the study.

4- Table 2: Clearly indicate that particular columns of p-values are for pairwise comparisons.

5- Include Supplemental Figure S1 as a standard figure within the manuscript.

**********

6. PLOS authors have the option to publish the peer review history of their article (what does this mean?). If published, this will include your full peer review and any attached files.

If you choose “no”, your identity will remain anonymous but your review may still be made public.

**Do you want your identity to be public for this peer review?** For information about this choice, including consent withdrawal, please see our Privacy Policy.

Reviewer #1: No

**********

[NOTE: If reviewer comments were submitted as an attachment file, they will be attached to this email and accessible via the submission site. Please log into your account, locate the manuscript record, and check for the action link "View Attachments". If this link does not appear, there are no attachment files.]

While revising your submission, please upload your figure files to the Preflight Analysis and Conversion Engine (PACE) digital diagnostic tool, https://pacev2.apexcovantage.com/. PACE helps ensure that figures meet PLOS requirements. To use PACE, you must first register as a user. Registration is free. Then, login and navigate to the UPLOAD tab, where you will find detailed instructions on how to use the tool. If you encounter any issues or have any questions when using PACE, please email PLOS at figures@plos.org. Please note that Supporting Information files do not need this step.

# Author comment: Author response to Decision Letter 0

**DOI:** 10.1371/journal.pone.0299802.r002

13 Dec 2022

Thank you for reviewing our manuscript and providing valuable feedback which we believe will strengthen the manuscript. We have responded to each of the reviewer’s comments in blue coloured font below. Changes in the manuscript have been tracked and page and line numbers have been provided. 

Journal Requirements:

When submitting your revision, we need you to address these additional requirements.

1. Please ensure that your manuscript meets PLOS ONE's style requirements, including those for file naming. The PLOS ONE style templates can be found at https://journals.plos.org/plosone/s/file?id=wjVg/PLOSOne_formatting_sample_main_body.pdf and 

https://journals.plos.org/plosone/s/file?id=ba62/PLOSOne_formatting_sample_title_authors_affiliations.pdf

Thank you for raising this. We have formatted the paper as per the journal requirements, including those for file naming. 

2. You indicated that you had ethical approval for your study. In your Methods section, please ensure you have also stated whether you obtained consent from parents or guardians of the minors included in the study or whether the research ethics committee or IRB specifically waived the need for their consent.

Thank you for this comment. We have added the following line to the ethical review section under the methods which states these women self-consented: 

“Women aged 16-17 were enrolled only in settings where permissible by national regulations and local ERC approval for their self-consent.” (Page 10, L206-208)

Reviewer #1: This paper reports secondary analysis of a large three-arm randomized trial. Women were randomized to DMPA-IM, the Cu-IUD, or LNG implant.

Minor revisions:

1- Abstract, Methods: The abstract states that the relative risk was estimated. List the events or provide a general term for events in which relative risks were estimated.

Thank you for noting this omission. The sentence in the abstract now reads (added text highlighted): 

Data collected for 12 to 18 months using 3-monthly behavioural questionnaires that relied on recall from the preceding 3 months, were used to estimate relative risk of sex behaviours between randomized groups using modified Poisson regression. (Page 4, L82-85)

2- Cite the statistical software used for the analysis.

Thank you for requesting this; we now cite the software, which was R version 4.1. (Page 7, L148-149)

3- Indicate the date range subjects participated in the study.

We have added the date range during which participants participated in the study, which was from 14 December 2015 to 31 October 2018. (Page 10, L211) 

4- Table 2: Clearly indicate that particular columns of p-values are for pairwise comparisons.

We have reorganized the table header to more clearly show that both RR/IRRs and p values arise from pairwise comparisons. (Pages 13-15)

5- Include Supplemental Figure S1 as a standard figure within the manuscript.

Thank you for this suggestion, we now include the figure as a standard figure in our manuscript. (Page 19)

Attachment Submitted filename: Response to Reviewers_13Dec22.pdf

# Peer review: Decision Letter 1

**DOI:** 10.1371/journal.pone.0299802.r003

**Authors:** Tulay Yilmaz

14 Mar 2023

PONE-D-22-20307R1Sexual behaviour among women using intramuscular depot medroxyprogesterone acetate, a copper intrauterine device, or a levonorgestrel implant for contraception: data from the ECHO randomized trialPLOS ONE

Dear Dr. Beesham,

Thank you for submitting your manuscript to PLOS ONE. After careful consideration, we feel that it has merit but does not fully meet PLOS ONE’s publication criteria as it currently stands. Therefore, we invite you to submit a revised version of the manuscript that addresses the points raised during the review process.

Please submit your revised manuscript by Apr 28 2023 11:59PM. If you will need more time than this to complete your revisions, please reply to this message or contact the journal office at plosone@plos.org. When you're ready to submit your revision, log on to https://www.editorialmanager.com/pone/ and select the 'Submissions Needing Revision' folder to locate your manuscript file.

Please include the following items when submitting your revised manuscript:

- A rebuttal letter that responds to each point raised by the academic editor and reviewer(s). You should upload this letter as a separate file labeled 'Response to Reviewers'.
- A marked-up copy of your manuscript that highlights changes made to the original version. You should upload this as a separate file labeled 'Revised Manuscript with Track Changes'.
- An unmarked version of your revised paper without tracked changes. You should upload this as a separate file labeled 'Manuscript'.

If you would like to make changes to your financial disclosure, please include your updated statement in your cover letter. Guidelines for resubmitting your figure files are available below the reviewer comments at the end of this letter.

If applicable, we recommend that you deposit your laboratory protocols in protocols.io to enhance the reproducibility of your results. Protocols.io assigns your protocol its own identifier (DOI) so that it can be cited independently in the future. For instructions see: https://journals.plos.org/plosone/s/submission-guidelines#loc-laboratory-protocols. Additionally, PLOS ONE offers an option for publishing peer-reviewed Lab Protocol articles, which describe protocols hosted on protocols.io. Read more information on sharing protocols at https://plos.org/protocols?utm_medium=editorial-email&utm_source=authorletters&utm_campaign=protocols.

We look forward to receiving your revised manuscript.

Kind regards,

Tulay Yilmaz, PhD

Academic Editor

PLOS ONE

Journal Requirements:

Please review your reference list to ensure that it is complete and correct. If you have cited papers that have been retracted, please include the rationale for doing so in the manuscript text, or remove these references and replace them with relevant current references. Any changes to the reference list should be mentioned in the rebuttal letter that accompanies your revised manuscript. If you need to cite a retracted article, indicate the article’s retracted status in the References list and also include a citation and full reference for the retraction notice.

[Note: HTML markup is below. Please do not edit.]

Reviewers' comments:

Reviewer's Responses to Questions

**Comments to the Author**

1. If the authors have adequately addressed your comments raised in a previous round of review and you feel that this manuscript is now acceptable for publication, you may indicate that here to bypass the “Comments to the Author” section, enter your conflict of interest statement in the “Confidential to Editor” section, and submit your "Accept" recommendation.

Reviewer #1: All comments have been addressed

Reviewer #2: All comments have been addressed

Reviewer #3: (No Response)

********** 2. Is the manuscript technically sound, and do the data support the conclusions?

The manuscript must describe a technically sound piece of scientific research with data that supports the conclusions. Experiments must have been conducted rigorously, with appropriate controls, replication, and sample sizes. The conclusions must be drawn appropriately based on the data presented. 

Reviewer #1: (No Response)

Reviewer #2: Yes

Reviewer #3: Yes

********** 3. Has the statistical analysis been performed appropriately and rigorously?

Reviewer #1: (No Response)

Reviewer #2: Yes

Reviewer #3: I Don't Know

********** 4. Have the authors made all data underlying the findings in their manuscript fully available?

The PLOS Data policy requires authors to make all data underlying the findings described in their manuscript fully available without restriction, with rare exception (please refer to the Data Availability Statement in the manuscript PDF file). The data should be provided as part of the manuscript or its supporting information, or deposited to a public repository. For example, in addition to summary statistics, the data points behind means, medians and variance measures should be available. If there are restrictions on publicly sharing data—e.g. participant privacy or use of data from a third party—those must be specified.

Reviewer #1: (No Response)

Reviewer #2: Yes

Reviewer #3: Yes

**********5. Is the manuscript presented in an intelligible fashion and written in standard English?

PLOS ONE does not copyedit accepted manuscripts, so the language in submitted articles must be clear, correct, and unambiguous. Any typographical or grammatical errors should be corrected at revision, so please note any specific errors here.

Reviewer #1: (No Response)

Reviewer #2: Yes

Reviewer #3: Yes

********** 6. Review Comments to the Author

Please use the space provided to explain your answers to the questions above. You may also include additional comments for the author, including concerns about dual publication, research ethics, or publication ethics. (Please upload your review as an attachment if it exceeds 20,000 characters)

Reviewer #1: (No Response)

Reviewer #2: I read this manuscript with great interest where the authors wanted to determined whether there were sexual behavioural differences amongst women on different contraceptives. To test this hypothesis would require a large sample number, which the authors have achieved through the ECHO trial. I have a few minor comments:

1. Could the authors please quote the ethics number of the study in the materials and methods.

2. Figure 1 is illegible and a higher resolution figure is required for publication purposes.

Reviewer #3: The study presents the results of secondary analysis of data collected as part of primary scientific research.

Results from the primary analysis of study data have been published; authors confirm results from secondary analysis reported have not been published elsewhere.

Unable to confirm whether experiments, statistics, and other analyses are performed to a high technical standard and are described in sufficient detail due to limited expertise in statistical analysis.

Conclusions are presented in an appropriate fashion and are supported by the data.

The article is presented in an intelligible fashion and is written in standard English.

The research meets all applicable standards for the ethics of experimentation and research integrity.

The article adheres to appropriate reporting guidelines and community standards for data availability.

**********7. PLOS authors have the option to publish the peer review history of their article (what does this mean?). If published, this will include your full peer review and any attached files.

If you choose “no”, your identity will remain anonymous but your review may still be made public.

**Do you want your identity to be public for this peer review?** For information about this choice, including consent withdrawal, please see our Privacy Policy.

Reviewer #1: No

Reviewer #2: **Yes: **Dr Michele Tomasicchio

Reviewer #3: **Yes: **Emma C. Aldrich

**********

[NOTE: If reviewer comments were submitted as an attachment file, they will be attached to this email and accessible via the submission site. Please log into your account, locate the manuscript record, and check for the action link "View Attachments". If this link does not appear, there are no attachment files.]

While revising your submission, please upload your figure files to the Preflight Analysis and Conversion Engine (PACE) digital diagnostic tool, https://pacev2.apexcovantage.com/. PACE helps ensure that figures meet PLOS requirements. To use PACE, you must first register as a user. Registration is free. Then, login and navigate to the UPLOAD tab, where you will find detailed instructions on how to use the tool. If you encounter any issues or have any questions when using PACE, please email PLOS at figures@plos.org. Please note that Supporting Information files do not need this step.

# Author comment: Author response to Decision Letter 1

**DOI:** 10.1371/journal.pone.0299802.r004

28 Apr 2023

Reviewer #2: I read this manuscript with great interest where the authors wanted to determined whether there were sexual behavioural differences amongst women on different contraceptives. To test this hypothesis would require a large sample number, which the authors have achieved through the ECHO trial

Response: Thank you

I have a few minor comments:

1. Could the authors please quote the ethics number of the study in the materials and methods.

Response: Thank you for raising this. We have added the ethics number to the methods section in the revised manuscript. The following text has been added:

“Ethical approval was obtained from the Protection of Human Subjects Committee of FHI 360 (approval number: 523201-146) and from the Ethics Committee (EC) of the WHO (approval numbers: A65897 and A65922). Each participating site also obtained approval from appropriate local Institutional Review Boards [12].” Page 10, Lines 203-206

2. Figure 1 is illegible and a higher resolution figure is required for publication purposes.

Response: This is noted. We have updated with a higher resolution image. Page 19. We have also uploaded the Figure as a separate attachment (not part of the word document), which does show a much better resolution of the figure. 

Reviewer #3: The study presents the results of secondary analysis of data collected as part of primary scientific research.

Results from the primary analysis of study data have been published; authors confirm results from secondary analysis reported have not been published elsewhere.

Unable to confirm whether experiments, statistics, and other analyses are performed to a high technical standard and are described in sufficient detail due to limited expertise in statistical analysis.

Conclusions are presented in an appropriate fashion and are supported by the data.

The article is presented in an intelligible fashion and is written in standard English.

The research meets all applicable standards for the ethics of experimentation and research integrity.

The article adheres to appropriate reporting guidelines and community standards for data availability.

Response: Thank you

# Peer review: Decision Letter 2

**DOI:** 10.1371/journal.pone.0299802.r005

**Authors:** Diwakar Mohan

12 Oct 2023

PONE-D-22-20307R2Sexual behaviour among women using intramuscular depot medroxyprogesterone acetate, a copper intrauterine device, or a levonorgestrel implant for contraception: data from the ECHO randomized trialPLOS ONE

Dear Dr. Beesham,

Thank you for submitting your manuscript to PLOS ONE. After careful consideration, we feel that it has merit but does not fully meet PLOS ONE’s publication criteria as it currently stands. Therefore, we invite you to submit a revised version of the manuscript that addresses the points raised during the review process.

==============================

Due to the discussions around the ECHO trial, we have tried to get more feedback for the manuscript. We have received feedback from another reviewer since your last response to reviewers. We would be grateful if you can address the new reviewer comments as well.Comments from editorI have a couple of points to make.1. There have been pairwise comparisons of each of the arms with a risk ratio and p value provided. I wonder about the need for such hypothesis testing since there are no base levels (true controls). Presenting just the n, incidence measure with 95% CI would be just as effective in providing the estimates from a well done RCT. As you have rightly pointed out multiple times, the large sample sizes and small event rates provide exaggerated risk ratios which may not be clinically significant.2. In the discussion, a mention has been made of the inability to comment on HIV acquisition using the present study. Has there been an effort to work with mediation analyses to provide more insight into the contribution of the changes in sexual behaviours towards HIV acquisition?

==============================

Please submit your revised manuscript by Nov 26 2023 11:59PM. If you will need more time than this to complete your revisions, please reply to this message or contact the journal office at plosone@plos.org. When you're ready to submit your revision, log on to https://www.editorialmanager.com/pone/ and select the 'Submissions Needing Revision' folder to locate your manuscript file.

Please include the following items when submitting your revised manuscript:

- A rebuttal letter that responds to each point raised by the academic editor and reviewer(s). You should upload this letter as a separate file labeled 'Response to Reviewers'.
- A marked-up copy of your manuscript that highlights changes made to the original version. You should upload this as a separate file labeled 'Revised Manuscript with Track Changes'.
- An unmarked version of your revised paper without tracked changes. You should upload this as a separate file labeled 'Manuscript'.

If you would like to make changes to your financial disclosure, please include your updated statement in your cover letter. Guidelines for resubmitting your figure files are available below the reviewer comments at the end of this letter.

If applicable, we recommend that you deposit your laboratory protocols in protocols.io to enhance the reproducibility of your results. Protocols.io assigns your protocol its own identifier (DOI) so that it can be cited independently in the future. For instructions see: https://journals.plos.org/plosone/s/submission-guidelines#loc-laboratory-protocols. Additionally, PLOS ONE offers an option for publishing peer-reviewed Lab Protocol articles, which describe protocols hosted on protocols.io. Read more information on sharing protocols at https://plos.org/protocols?utm_medium=editorial-email&utm_source=authorletters&utm_campaign=protocols.

We look forward to receiving your revised manuscript.

Kind regards,

Diwakar Mohan, MD

Academic Editor

PLOS ONE

[Note: HTML markup is below. Please do not edit.]

Reviewers' comments:

Reviewer's Responses to Questions

**Comments to the Author**

1. If the authors have adequately addressed your comments raised in a previous round of review and you feel that this manuscript is now acceptable for publication, you may indicate that here to bypass the “Comments to the Author” section, enter your conflict of interest statement in the “Confidential to Editor” section, and submit your "Accept" recommendation.

Reviewer #2: All comments have been addressed

Reviewer #4: All comments have been addressed

**********

2. Is the manuscript technically sound, and do the data support the conclusions?

The manuscript must describe a technically sound piece of scientific research with data that supports the conclusions. Experiments must have been conducted rigorously, with appropriate controls, replication, and sample sizes. The conclusions must be drawn appropriately based on the data presented. 

Reviewer #2: Yes

Reviewer #4: Partly

**********

3. Has the statistical analysis been performed appropriately and rigorously? 

Reviewer #2: Yes

Reviewer #4: No

**********

4. Have the authors made all data underlying the findings in their manuscript fully available?

The PLOS Data policy requires authors to make all data underlying the findings described in their manuscript fully available without restriction, with rare exception (please refer to the Data Availability Statement in the manuscript PDF file). The data should be provided as part of the manuscript or its supporting information, or deposited to a public repository. For example, in addition to summary statistics, the data points behind means, medians and variance measures should be available. If there are restrictions on publicly sharing data—e.g. participant privacy or use of data from a third party—those must be specified.

Reviewer #2: Yes

Reviewer #4: No

**********

5. Is the manuscript presented in an intelligible fashion and written in standard English?

PLOS ONE does not copyedit accepted manuscripts, so the language in submitted articles must be clear, correct, and unambiguous. Any typographical or grammatical errors should be corrected at revision, so please note any specific errors here.

Reviewer #2: Yes

Reviewer #4: Yes

**********

6. Review Comments to the Author

Please use the space provided to explain your answers to the questions above. You may also include additional comments for the author, including concerns about dual publication, research ethics, or publication ethics. (Please upload your review as an attachment if it exceeds 20,000 characters)

Reviewer #2: (No Response)

Reviewer #4: Thank you for the opportunity to review this very interesting paper. The paper is generally well written and addresses an important issue in public health- the effects of contraceptives on sexual desire (mood), adverse events and sexual behavior. The authors used data from the ECHO trial which has courted controversy since its design, conduct and dissemination of results. While a lot of the controversy regarding DMPA-IM and HIV acquisition has decreased, this contraceptive method still carries stigma of forced use, lack of education and options. The authors need to make it clear what their hypothesis going into the study was and better discuss what the implications of these findings are.

I have made more specific comments by section

• Abstract, background: the authors need to add a hypothesis for this analysis. I am struggling to understand why this analysis is necessary given that the data used is from a randomized trial. If the randomization at baseline worked well, should we expect differences in sexual behavior, libido, bleeding and sexual behavior by arm?

• Abstract, methods: The authors need to specify the sexual behaviors in question

• Abstract, results: The authors report that DMPA-IM was associated with lower sexual behaviors. I am not sure if lower is the correct term. Lower prevalence of specified high risk sexual behaviours maybe would be better

• Abstract results: Are libido and menstrual disturbances sexual behaviours? I would have thought these would be adverse effects of the contraception and would be expected to explain some of the sexual behaviours then observed. This is the reason I am asking the authors to state a hypothesis and add libido and menstrual disturbances as objectives/ outcomes in the analysis.

• Can the authors also present the relative risks in addition to the raw percentages

Introduction

• Line 133- can the authors specify the behaviors in question and state the hypothesis

Methods

• Line 151- 157: see earlier comment regarding side effects vs behaviors

• Line 171: Did the authors also consider adjusting for age, living with partner and any other variables for which there appeared to be imbalance at baseline e.g. new partner at baseline, and more than one partner in the last 3 months at baseline

Results

Table 1-

• please present baseline data on menstrual disturbances if that data is available

• Are the differences in any new partner and more than one sex partner by arm significant?

Table 2:

• in the main analysis, a 50% difference in HIV incidence was the threshold for recommending policy change, is there such a threshold for the differences in adverse events such as menstrual disturbances, libido and sexual behavior

• Indicate that RR are adjusted

Figure 1

• Plot also menstrual disturbances and libido

Discussion

• Was there any qualitative work done in this trial or elsewhere on the psychological effects of absent or irregular menstruation could explain effects of DMPA-IM on libido and sexual behaviors.

• I feel that the so what of this paper needs to be strengthened. What are we saying to women who opt for DMPA-IM and the health care providers that provide it?

**********

7. PLOS authors have the option to publish the peer review history of their article (what does this mean?). If published, this will include your full peer review and any attached files.

If you choose “no”, your identity will remain anonymous but your review may still be made public.

**Do you want your identity to be public for this peer review?** For information about this choice, including consent withdrawal, please see our Privacy Policy.

Reviewer #2: No

Reviewer #4: No

**********

[NOTE: If reviewer comments were submitted as an attachment file, they will be attached to this email and accessible via the submission site. Please log into your account, locate the manuscript record, and check for the action link "View Attachments". If this link does not appear, there are no attachment files.]

While revising your submission, please upload your figure files to the Preflight Analysis and Conversion Engine (PACE) digital diagnostic tool, https://pacev2.apexcovantage.com/. PACE helps ensure that figures meet PLOS requirements. To use PACE, you must first register as a user. Registration is free. Then, login and navigate to the UPLOAD tab, where you will find detailed instructions on how to use the tool. If you encounter any issues or have any questions when using PACE, please email PLOS at figures@plos.org. Please note that Supporting Information files do not need this step.

# Author comment: Author response to Decision Letter 2

**DOI:** 10.1371/journal.pone.0299802.r006

18 Dec 2023

Due to the discussions around the ECHO trial, we have tried to get more feedback for the manuscript. We have received feedback from another reviewer since your last response to reviewers. We would be grateful if you can address the new reviewer comments as well.

This is noted. Please find responses to editor’s and reviewers’ comments below. We have provided page and line numbers in the revised manuscript where indicated. 

Comments from editor

I have a couple of points to make.

1. There have been pairwise comparisons of each of the arms with a risk ratio and p value provided. I wonder about the need for such hypothesis testing since there are no base levels (true controls). Presenting just the n, incidence measure with 95% CI would be just as effective in providing the estimates from a well done RCT. As you have rightly pointed out multiple times, the large sample sizes and small event rates provide exaggerated risk ratios which may not be clinically significant.

Thank you for this comment. We very much agree with the editor that the descriptive data should be emphasized, so that statistical significance is not taken to imply clinical significance for participants. This is why, given limited space in the abstract, we give descriptive statistics of each outcome by randomized group and not the risk ratios. 

We submit that the large sample size and small event rates do not yield exaggerated risk ratios (risk ratios would not be expected to be biased by either large sample sizes or small event rates); our intention was to point out that the large sample size means we can find statistically significant differences which are small enough that they are not clinically significant for participants. We reviewed our statements carefully to ensure we do not imply bias, in Lines 207-209 (Page 10):

“However, in a study of 7829 women powered to detect differences in HIV acquisition, we likely had very high power to detect even small differences which may not necessarily be clinically significant.” 

And Lines 247-248 (Page 13):

“Many comparisons were statistically significant, even when differences were small, as expected when comparing large groups.”

Regarding whether RRs should be computed at all, we don’t agree that only a ‘true control’ comparator would justify hypothesis testing. We think that comparison across different highly effective contraceptive methods is an important study question and does justify hypothesis testing. Our hypothesis, which perhaps we have not been clear enough in stating (see our response to reviewer 2, below) is that there are differences among the studied highly effective contraceptive choices regarding how they affect sexual behaviours and related outcomes. We believe this is important, particularly for women who want highly effective contraceptive options and may benefit from information to inform choices. We believe we have balanced using statistical testing with careful interpretation and highlighting the numbers compared rather than just RRs.

2. In the discussion, a mention has been made of the inability to comment on HIV acquisition using the present study. Has there been an effort to work with mediation analyses to provide more insight into the contribution of the changes in sexual behaviours towards HIV acquisition?

The primary publication of the ECHO trial presented secondary analyses comparing HIV acquisition by randomized group using causal methods. The causal models were conducted in two ways; first, using models did not adjust for any follow-up covariates, and second, adjusting for post-baseline sex behaviours of condomless sex, having a new partner, and having multiple partners (Table S10 in the primary publication’s supplement, and provided below) Comparison of results of these models (rows 2 and 3 below) shows that the adjustment for post-baseline differences in sex behaviors, which is what we report on in the current manuscript, made little to no difference to relative risks of HIV acquisition between groups. This implies that observed differences in sex behaviors caused by differing contraceptive methods do not translate to differences in HIV risk. We now explain this more clearly in the discussion under Relevance to HIV acquisition: (Page 21, Lines 370-377):

“Whether the small but generally consistent differences in sexual behaviour may have contributed to the overall similar HIV risk among the three methods found in the ECHO Trial cannot be confirmed from this analysis. Secondary comparisons of HIV incidence among the three contraceptive methods using causal models showed essentially no effect from adjusting for follow-up behaviours of condomless sex, having a new partner or having multiple partners on comparisons between randomized contraceptive method, implying that the differences in those behaviours by method do not contribute to differences in HIV risk by method [12].” (added text in bold)

For reference, Table S10 from the ECHO primary publication: 

 DMPA-IM vs Copper IUD DMPA-IM vs LNG mplant Copper IUD vs LNG Implant

Analysis HR (96% CI) p-value HR (96% CI) p-value HR (96% CI) p-value

Unweighted, adjusted for baseline covariates 1.17 (0.89, 1.53) 0.25 1.34 (1.02, 1.77) 0.029 1.15 (0.88, 1.51) 0.29

Weighted, adjusted for baseline covariates 1.07 (0.82, 1.40) 0.62 1.27 (0.96, 1.68) 0.080 1.19 (0.91, 1.56) 0.19

Weighted, adjusted for baseline and time-varying covariates 1.10 (0.84, 1.44) 0.49 1.29 (0.97, 1.71) 0.060 1.18 (0.90, 1.55) 0.22

Continuous use estimates included only time on study with continuous use of randomized methods, as defined in the Methods, and included data from 7317 women. Causal analysis methods were used to estimate HRs during continuous use, with inverse probability weighting for discontinuation of randomized method, adjusted for baseline covariates (age ≤24, having living children, living with husband or primary partner, vaginal sex without a condom, more than one sex partner, and new sex partner) and time-varying covariates (vaginal sex without a condom, more than one sex partner and new sex partner in the previous 3 months). Inverse probability weights for discontinuation of randomized method relied on vaginal sex without a condom, vaginal sex last 7 days, and having primary partner in the previous 3 months. Covariate data for calculating weights were missing for 82 (1.1%) of women; and covariates for model adjustment were missing for another 9 (0.1%) of women, corresponding to 0.1% of visits. Women missing censoring weights or covariate data were excluded from the causal analysis.

Comments from reviewers

Reviewer #2: (No Response)

Noted. 

Reviewer #4: Thank you for the opportunity to review this very interesting paper. The paper is generally well written and addresses an important issue in public health- the effects of contraceptives on sexual desire (mood), adverse events and sexual behavior. The authors used data from the ECHO trial which has courted controversy since its design, conduct and dissemination of results. While a lot of the controversy regarding DMPA-IM and HIV acquisition has decreased, this contraceptive method still carries stigma of forced use, lack of education and options. The authors need to make it clear what their hypothesis going into the study was and better discuss what the implications of these findings are.

Thank you for these comments. We have defined our hypothesis more clearly, and the implications of our study findings in the comments below. 

I have made more specific comments by section.

• Abstract, background: the authors need to add a hypothesis for this analysis. I am struggling to understand why this analysis is necessary given that the data used is from a randomized trial. If the randomization at baseline worked well, should we expect differences in sexual behavior, libido, bleeding and sexual behavior by arm?

The reason we might expect to find differences is that DMPA and Levonorgestrel have large and different effects on endogenous hormone levels including estradiol, testosterone and sex hormone binding globulin, which are associated with sexual function and mood. Our hypothesis was that there may be differences in sexual activity across different highly effective contraceptive methods studied. We believe this is of interest for women making choices among contraceptive options. We have edited the abstract to explain our study aim more clearly (Page 4, Lines 73-76):

“We set out to obtain robust evidence on the relative effects of three contraceptive methods on sex behaviours, which is important for guiding contraceptive choice and future contraceptive developments.” (added text in bold)

Regarding why we set out to compare sexual differences in a randomized trial, the purpose of randomization is to obtain comparable groups at baseline, for both measured and unmeasured factors. There is no reason to expect that measurements after baseline (i.e., once on the different treatments) will not differ between groups depending on the effect of the treatment(s) on the outcome(s) of interest, and this is what we set out to examine. As stated in the methods (Page 8, Lines 168-172):

“To compare each behaviour by randomized group in an overall comparison, we made pairwise comparisons between randomized groups of each outcome in an intention-to-treat (ITT) analysis, meaning women’s visits were analysed according to randomized assignment rather than by whether they were adhering to their randomized method. We used all available post-baseline observations.” (bold added here for emphasis)

We have now specified this more clearly in the abstract (Page 4, Lines 83-86):

“Data collected for 12 to 18 months using 3-monthly behavioural questionnaires that relied on recall from the preceding 3 months, were used to estimate relative risk of post-baseline sex behaviours, as well as sexual desire and menstrual bleeding between randomized groups using modified Poisson regression.” (relevant new text in bold)

And, at the start of the methods (Page 7, Lines 149-151)

“Our primary interest was to compare post-randomization sex behaviours and related outcomes by randomization group, overall and over time. The analysis plan for this work was completed before starting the analyses.” (relevant new text in bold)

• Abstract, methods: The authors need to specify the sexual behaviors in question 

While we’d like to list these in the abstract methods as well as in the results, we examined a variety of sexual behaviours, and it would make the abstract considerably longer to list the sexual behaviours we examined in both methods and when providing the results. However, to improve clarity in the methods we now clarify that we also examined sexual desire and menstrual bleeding, as the reviewer’s point elsewhere that these are not sex behaviours, is well taken. (Page 4, Lines 83-86)

“Data collected for 12 to 18 months using 3-monthly behavioural questionnaires that relied on recall from the preceding 3 months, were used to estimate relative risk of post-baseline sex behaviours, as well as sexual desire and menstrual bleeding between randomized groups using modified Poisson regression.” (relevant new text in bold)

• Abstract, results: The authors report that DMPA-IM was associated with lower sexual behaviors. I am not sure if lower is the correct term. Lower prevalence of specified high risk sexual behaviours maybe would be better.

This point is noted. We have edited as suggested (Page 4, Lines 88-90):

“We observed small but generally consistent effects wherein DMPA-IM users reported lower prevalence of specified high risk sexual behaviours than implant users than Cu-IUD users …” (relevant new text in bold)

• Abstract results: Are libido and menstrual disturbances sexual behaviours? I would have thought these would be adverse effects of the contraception and would be expected to explain some of the sexual behaviours then observed. This is the reason I am asking the authors to state a hypothesis and add libido and menstrual disturbances as objectives/ outcomes in the analysis.

We appreciate this point and now specify “sex behaviours, sexual desire and menstrual bleeding” in the abstract methods. (Page 4, Lines 83-86)

“Data collected for 12 to 18 months using 3-monthly behavioural questionnaires that relied on recall from the preceding 3 months, were used to estimate relative risk of post-baseline sex behaviours, as well as sexual desire and menstrual bleeding between randomized groups using modified Poisson regression.” (new text in bold)

We also have moved the results of these two outcomes to a separate sentence rather than including them in the sentence giving results for sex behaviours: (Pages 4-5, Lines 88-99)

“We observed small but generally consistent effects wherein DMPA-IM users had reported lower prevalence of specified high risk sexual behaviours than implant users than Cu-IUD users (the ‘>’ and ‘<’ symbols indicate statistically significant differences): multiple sex partners 3.6% < 4.8% < 6.2% respectively; new sex partner 3.0% < 4.0% <5.3%; coital acts 16.45, 16.65, 17.12 (DMPA-IM < Cu-IUD); unprotected sex 65% < 68%, 70%; unprotected sex past 7 days 33% <36%, 37%; sex during vaginal bleeding 7.1%, 7.1% < 8.9%; no sex acts 4.1%, 3.8%, 3.4% (DMPA-IM > Cu-IUD); partner has sex with others 10% < 11%, 11%. The one exception was having any sex partner 96.5%, 96.9% <97.4% (DMPA-IM < Cu-IUD). Decrease in sexual desire was reported by 1.6% > 1.1% >0.5%; amenorrhoea by 49% > 41% >12% and regular menstrual pattern by 26% <35% <87% respectively.” (relevant new text in bold)

• Can the authors also present the relative risks in addition to the raw percentages 

While we would have liked to present the relative risks in the abstract in addition to the raw percentages, with so many outcomes of interest, and with 3 pairwise comparisons for each one, with limited abstract space, we have had to limit the detail reported for each comparison. We are also cognizant of the editor’s point#1 asking us to emphasize descriptives over relative risks. Therefore, while we appreciate the reviewer’s interest in seeing these highlighted, we have kept the text as is for the abstract.

Introduction

• Line 133- can the authors specify the behaviors in question and state the hypothesis

We now state the hypothesis of the current analysis in the final sentence of the introduction, as follows: (Page 7, Lines 134-136)

“The current analysis tests whether longitudinal sex behaviours, sexual desire or menstrual pattern differs in women using the three methods of contraception offered in the ECHO Trial.” (new text in bold)

Thank you for your query about specifying the behaviours tested. We listed the outcomes in the methods under “Outcomes”, but the list is now more precisely detailed for clarity: (Page 8, Lines 154-164)

“Sex behaviour outcomes identified a priori were self-reported (for the past 3 months, unless otherwise specified): having any sex partner, having multiple sex partners, having a new sex partner, number of coital acts (continuous variable), sex during a time of vaginal bleeding, any unprotected sex, and, for the past 7 days, number of unprotected sex acts (both as a continuous variable and dichotomized as any unprotected sex). We also analyzed no sex acts, partner having sex with other partners, and measures of side effects: reduced sexual desire, regular menstruation and absence of menstruation.” (relevant new text in bold)

Checking Table 2 (Pages 14-16), we found that Table 2 erroneously reported the same outcome twice under two different names, “Any unprotected sex acts” and “sex but not always used condom”. We have removed the latter from Table 2 to avoid redundancy. We have also revised the labelling in Table 2 to avoid calling all outcomes ‘Behaviours” and now separate those that are side effects or a behaviour of the woman’s partner, not the woman. 

Methods:

• Line 151- 157: see earlier comment regarding side effects vs behaviors

See response to immediately prior to this comment. 

• Line 171: Did the authors also consider adjusting for age, living with partner and any other variables for which there appeared to be imbalance at baseline e.g. new partner at baseline, and more than one partner in the last 3 months

Attachment Submitted filename: Responses to reviewers_Sex Beh manuscript_18Dec23.pdf

# Peer review: Decision Letter 3

**DOI:** 10.1371/journal.pone.0299802.r007

**Authors:** Diwakar Mohan

16 Feb 2024

Sexual behaviour among women using intramuscular depot medroxyprogesterone acetate, a copper intrauterine device, or a levonorgestrel implant for contraception: data from the ECHO randomized trial

PONE-D-22-20307R3

Dear Dr. Beesham,

We’re pleased to inform you that your manuscript has been judged scientifically suitable for publication and will be formally accepted for publication once it meets all outstanding technical requirements.

Within one week, you’ll receive an e-mail detailing the required amendments. When these have been addressed, you’ll receive a formal acceptance letter and your manuscript will be scheduled for publication.

An invoice for payment will follow shortly after the formal acceptance. To ensure an efficient process, please log into Editorial Manager at http://www.editorialmanager.com/pone/, click the 'Update My Information' link at the top of the page, and double check that your user information is up-to-date. If you have any billing related questions, please contact our Author Billing department directly at authorbilling@plos.org.

If your institution or institutions have a press office, please notify them about your upcoming paper to help maximize its impact. If they’ll be preparing press materials, please inform our press team as soon as possible -- no later than 48 hours after receiving the formal acceptance. Your manuscript will remain under strict press embargo until 2 pm Eastern Time on the date of publication. For more information, please contact onepress@plos.org.

Kind regards,

Diwakar Mohan, MD

Academic Editor

PLOS ONE

Additional Editor Comments (optional):

Reviewers' comments:

Reviewer's Responses to Questions

**Comments to the Author**

1. If the authors have adequately addressed your comments raised in a previous round of review and you feel that this manuscript is now acceptable for publication, you may indicate that here to bypass the “Comments to the Author” section, enter your conflict of interest statement in the “Confidential to Editor” section, and submit your "Accept" recommendation.

Reviewer #4: All comments have been addressed

**********

2. Is the manuscript technically sound, and do the data support the conclusions?

The manuscript must describe a technically sound piece of scientific research with data that supports the conclusions. Experiments must have been conducted rigorously, with appropriate controls, replication, and sample sizes. The conclusions must be drawn appropriately based on the data presented. 

Reviewer #4: Yes

**********

3. Has the statistical analysis been performed appropriately and rigorously? 

Reviewer #4: Yes

**********

4. Have the authors made all data underlying the findings in their manuscript fully available?

The PLOS Data policy requires authors to make all data underlying the findings described in their manuscript fully available without restriction, with rare exception (please refer to the Data Availability Statement in the manuscript PDF file). The data should be provided as part of the manuscript or its supporting information, or deposited to a public repository. For example, in addition to summary statistics, the data points behind means, medians and variance measures should be available. If there are restrictions on publicly sharing data—e.g. participant privacy or use of data from a third party—those must be specified.

Reviewer #4: No

**********

5. Is the manuscript presented in an intelligible fashion and written in standard English?

PLOS ONE does not copyedit accepted manuscripts, so the language in submitted articles must be clear, correct, and unambiguous. Any typographical or grammatical errors should be corrected at revision, so please note any specific errors here.

Reviewer #4: Yes

**********

6. Review Comments to the Author

Please use the space provided to explain your answers to the questions above. You may also include additional comments for the author, including concerns about dual publication, research ethics, or publication ethics. (Please upload your review as an attachment if it exceeds 20,000 characters)

Reviewer #4: Thank you for the opportunity to re-review this revised manuscript. I found that the authors carefully responded and addressed all the comments I had on the previous version of the manuscript. I have no further comments

**********

7. PLOS authors have the option to publish the peer review history of their article (what does this mean?). If published, this will include your full peer review and any attached files.

If you choose “no”, your identity will remain anonymous but your review may still be made public.

**Do you want your identity to be public for this peer review?** For information about this choice, including consent withdrawal, please see our Privacy Policy.

Reviewer #4: **Yes: **Tendesayi Kufa

**********

# Editor report: Acceptance letter

**DOI:** 10.1371/journal.pone.0299802.r008

**Authors:** Diwakar Mohan

26 Apr 2024

PONE-D-22-20307R3 

PLOS ONE

Dear Dr. Beesham, 

I'm pleased to inform you that your manuscript has been deemed suitable for publication in PLOS ONE. Congratulations! Your manuscript is now being handed over to our production team.

At this stage, our production department will prepare your paper for publication. This includes ensuring the following:

* All references, tables, and figures are properly cited

* All relevant supporting information is included in the manuscript submission,

* There are no issues that prevent the paper from being properly typeset

If revisions are needed, the production department will contact you directly to resolve them. If no revisions are needed, you will receive an email when the publication date has been set. At this time, we do not offer pre-publication proofs to authors during production of the accepted work. Please keep in mind that we are working through a large volume of accepted articles, so please give us a few weeks to review your paper and let you know the next and final steps. 

Lastly, if your institution or institutions have a press office, please let them know about your upcoming paper now to help maximize its impact. If they'll be preparing press materials, please inform our press team within the next 48 hours. Your manuscript will remain under strict press embargo until 2 pm Eastern Time on the date of publication. For more information, please contact onepress@plos.org.

If we can help with anything else, please email us at customercare@plos.org.

Thank you for submitting your work to PLOS ONE and supporting open access. 

Kind regards, 

PLOS ONE Editorial Office Staff

on behalf of

Dr. Diwakar Mohan 

Academic Editor

PLOS ONE
